# Engineered microbes to enable a circular economy for biodegradable plastics

**DOI:** 10.3389/fmicb.2026.1828166

**Published:** 2026-07-02

**Authors:** Dushmantha Madushanka, Cole Beard, Bhagya S. Kolitha, Lakshika Dissanayake, Sandhya Jayasekara, Saptarshi Ghosh, Lahiru N. Jayakody

**Affiliations:** School of Biological Sciences, Southern Illinois University, Carbondale, IL, United States

**Keywords:** biodegradable plastics, bio-upcycling, circular economy, metabolic engineering, synthetic biology

## Abstract

Plastic pollution resulting from the continued dominance of fossil-derived polymers is a major global environmental challenge. Although biodegradable plastics, such as polylactic acid (PLA), polyhydroxyalkanoates (PHAs), polybutylene succinate (PBS), polybutylene adipate terephthalate (PBAT), and related materials, are increasingly being deployed as alternatives, their environmental performance is frequently constrained by infrastructure gaps, uncontrolled carbon loss, and incomplete degradation under realistic conditions. Therefore, biodegradability alone does not guarantee circularity of the material. To address this, intentional rerouting of plastics and their monomers into upcycling streams offers a widely applicable solution. This review advances the circular bioeconomy framework built on engineered depolymerization and metabolic bio-funneling of biodegradable and selected synthetic plastics. We present recent progress in enzyme-mediated polyester breakdown, emphasizing hydrolases and oxidoreductases, the kinetic and structural determinants of activity, and protein engineering strategies that broaden substrate scope and enhance operational stability. We then organize bio-upcycling strategies according to key metabolic entry nodes: pyruvate, acetyl-CoA/β-oxidation, and aromatic/dicarboxylate pathways, to demonstrate how plastic-derived monomers can be systematically redirected toward platform chemicals, fuels, specialty monomers, and next-generation biopolymers through pathway rewiring, flux control, and redox balance. In addition to biological conversion, we evaluate chemo-biological hybrid systems and integrated techno-economic and life cycle considerations, including process efficiency, enzyme cost, toxicity mitigation, and infrastructure compatibility. We further highlight emerging tools, such as systems biology, adaptive laboratory evolution, synthetic consortia design, and machine-learning-guided protein optimization, which accelerate the design–build–test–learn cycle for scalable microbial platforms for plastic upcycling. Collectively, this study reframes biodegradable plastics not as materials designed merely to disappear but as programmable carbon reservoirs that can be captured, upgraded, and reintegrated into biomanufacturing value chains. Actively closing the loop through engineered bio-upcycling, rather than relying on passive environmental degradation, offers a practical pathway to align plastic utility with environmental sustainability and achieve a truly circular bioeconomy.

## Graphical abstract

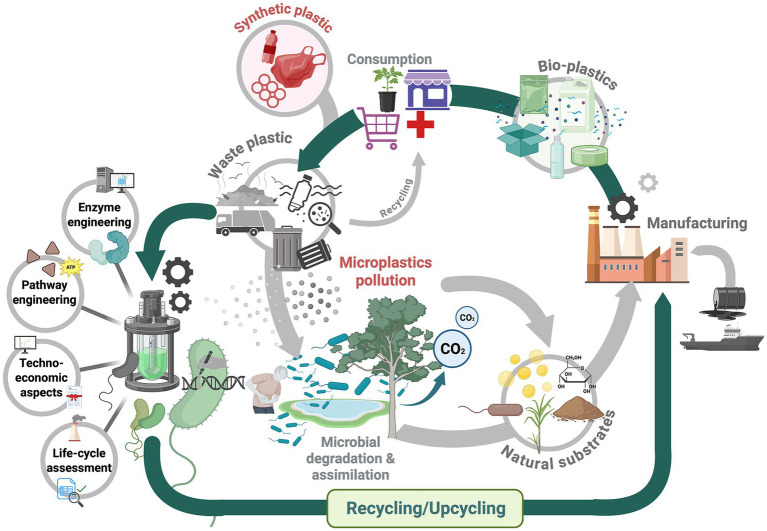

## 1 Introduction

More than 450 million metric tons of plastic are produced annually, with most plastic entering a linear “take-make-dispose” model (Sonke et al., 2025). Only 9–14% of plastic is recycled due to limitations of technologically feasible processes; most is landfilled (~50%), incinerated (~19%), or mismanaged. Petroleum-derived micro- and nanoplastics accumulate in soils, freshwater, marine environments, and the atmosphere, posing risks to both ecological and human health (Zhang et al., 2022; Détrée and Gallardo-Escárate, 2018; Chiu et al., 2015). The accumulation of synthetic plastics in ecosystems is a critical environmental crisis (Macreadie and Adyel, 2025). Low recycled feedstocks in the current plastic industry reflect the continued dominance of virgin fossil-based materials and their limited circularity (Houssini et al., 2025). At current rates, plastics could use up to 20% of the global carbon budget within 15 years (Zheng and Suh, 2019). These trends underscore the urgency of developing eco-friendly and sustainable materials and systems that enable carbon circularity rather than continued environmental dispersion (Jin et al., 2024; Lakhiar et al., 2024; Brahney et al., 2020).

Biodegradable plastics, including polylactic acid (PLA), polyhydroxyalkanoates (PHAs), polybutylene succinate (PBS), polybutylene adipate terephthalate (PBAT), and polycaprolactone (PCL), have emerged as promising alternatives to synthetic plastics (Hwang et al., 2025). Many are bio-based or partially renewable, whereas others incorporate fossil fuel inputs but with enhanced degradability. Microorganisms break them down into CO_2_, CH_4_, and microbial biomass, although the rates depend on environmental conditions. Biodegradable plastics are especially valuable when recycling or reuse is not possible, such as in single-use applications (Samir et al., 2022). However, this end-of-life advantage is conditional on two factors. First, when biodegradable polymers are mixed into conventional plastic recycling streams, they can act as contaminants that compromise the quality of the recycled resin. Therefore, their value depends on dedicated collection and the availability of appropriate composting or upcycling infrastructure. Second, in real environments, such as soil and marine settings, the conditions required for complete mineralization, including suitable temperature, moisture, oxygen, and microbial communities, are often lacking (Kim M. S. et al., 2023). Intrinsic material properties, such as high crystallinity, large particle size, aromatic units (e.g., terephthalate), hydrophobic additives, and plasticizers, reduce enzyme accessibility and slow biodegradation (Olaiya et al., 2019). This leads to incomplete and prolonged degradation, leaving persistent micro- and nanoparticles. Notably, biodegradable microplastics have a higher sorption capacity for micropollutants than synthetic microplastics (Cao et al., 2024). Intermediate degradation products, such as plasticizers and stabilizers, secondary chemical transformations, such as chlorination, and CH_4_ released under unmanaged conditions, contribute to ecotoxicity, ecosystem disruption, and greenhouse gas emissions during the end-of-life phase, despite being labeled as “biodegradable” (Molina-Besch, 2022). Consistent with these findings, *in vitro* toxicity screening has shown that several commercial bioplastics and plant-based products can elicit toxicity comparable to that of conventional plastics, although hazard profiles vary substantially across products and exposure conditions (Zimmermann et al., 2020). Most biodegradable polymers (e.g., PLA, PBAT, and PBS) require industrial composting conditions with high temperatures, controlled moisture, and specific microorganisms/enzymes, and do not degrade efficiently in ambient soil, water, or marine environments (Gu, 2021). Therefore, designing a polymer to degrade and a material system to be circular are distinct objectives: the first addresses disappearance, the second addresses recovery, and biodegradability alone does not deliver circularity.

A circular bioeconomy framework ensures that both biodegradable and synthetic plastics are routed through designed depolymerization and microbial valorization systems rather than through unmanaged decay (Johansen et al., 2022). In these systems, polymer carbon is enzymatically or chemically released as defined monomers and funneled through engineered metabolic pathways to regenerate polymers or to generate high-value chemicals (Sullivan et al., 2022). Synthetic microbial consortia can further expand the substrate scope to include recalcitrant synthetic polymers and integrate them into closed-loop biodegradable polymer production systems. Hence, despite this review’s central focus on biodegradable plastics, we deliberately included selected synthetic polyesters (most notably PET and to a lesser extent PEF) as benchmark systems, as they release monomers (terephthalate, ethylene glycol, and FDCA) that converge on the same metabolic node as biodegradable plastic monomers and are therefore amenable to the same engineered upcycling logic. These bio-recycling platforms transform linear plastic waste streams into regenerative value chains, creating economic incentives for recovery and management while reducing environmental impact (Jayakody et al., 2024). By reframing biodegradable plastic waste as a renewable carbon feedstock rather than as an environmental liability, this approach integrates biological catalysis with process engineering to close material loops and offers environmental and economic benefits. Unlike mechanical recycling, where polymer chain scission and additive carry-over progressively reduce mechanical performance, monomer-level bio-recirculation can, in principle, support the resynthesis of virgin-equivalent polymers, provided that the recovered monomers reach polymerization-grade purity and that downstream process economics are competitive (Haq et al., 2025).

**Figure 1:**
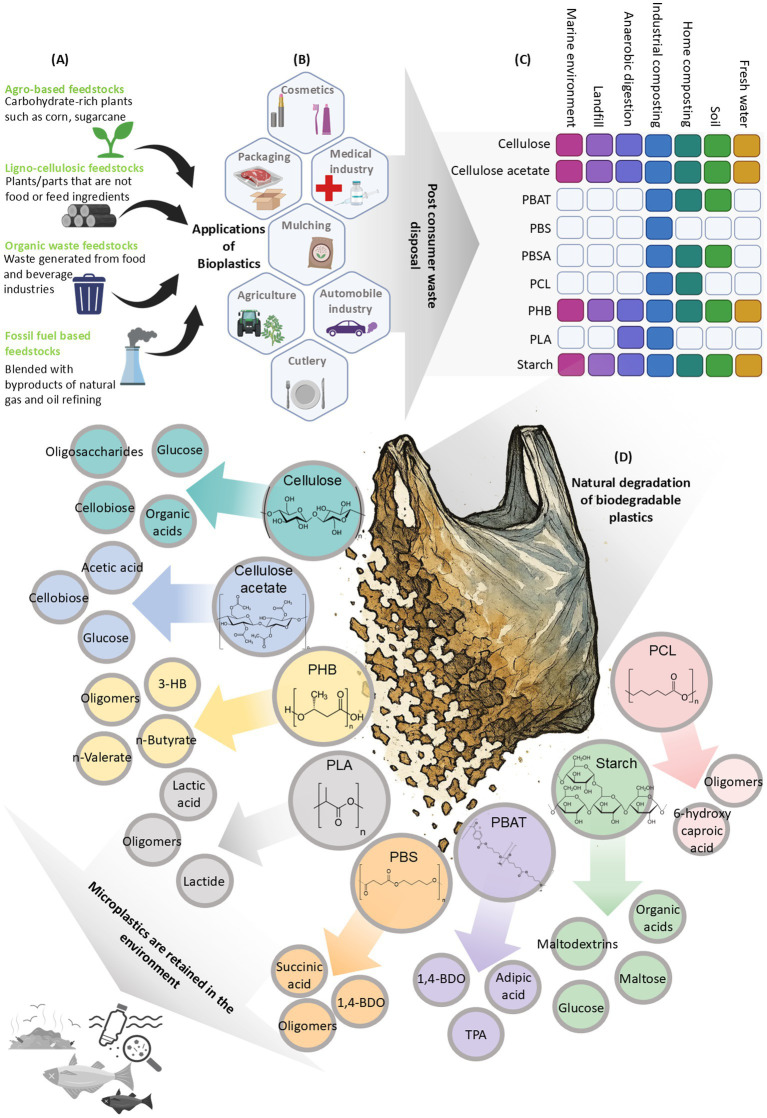
Overview of the production, applications, biodegradability, and degradation products of different biodegradable polymer materials. **(A)** Feedstocks are used to produce biodegradable polymers. Biodegradable polymer materials are produced using natural, renewable, and organic waste, as well as oil refinery-based raw materials. **(B)** Applications of biodegradable plastics. Biodegradable plastics are used in a variety of applications, from packaging to biomedical uses, replacing non-biodegradable plastics such as polyethylene terephthalate (PET), polyvinyl chloride (PVC), polystyrene (PS), and polypropylene (PP) plastics. **(C)** Overview of biodegradation and the conditions required for the biodegradation of biodegradable plastics. The solid colors indicate proven biodegradability or proven biodegradability under certain conditions or at least certain grades of polymer type. Shaded cells indicate an environment in which the polymer is not biodegraded. The conditions specified for the respective environments and pass/fail criteria were as follows: marine environment (30 °C, 90% biodegradation within a maximum of 6 months); fresh water (21 °C, 90% biodegradation within a maximum of 56 days); soil (25 °C, 90% biodegradation within a maximum of 2 years); home composting (28 °C, 90% biodegradation within a maximum of 12 months); landfill (no standard specifications or certification scheme available since it is not considered a preferred end-of-life option); anaerobic digestion (thermophilic 52 °C/mesophilic 37 °C, standard specification not yet available, but 90% generally considered as completely biodegradable); and industrial composting (58 °C, 90% biodegradation within a maximum of 6 months) (Yu and Flury, 2024). **(D)** Schematic overview of biodegradation products generated during enzymatic biodegradation of different polymers present in biodegradable plastics, including monomeric and oligomeric intermediates, such as lactic acid, lactide, 3-hydroxybutyrate, succinic acid, 1,4-butanediol, terephthalic acid, adipic acid, 6-hydroxycaproic acid, and oligosaccharide/glucose mixtures, as well as the short-chain platform acids n-butyrate and n-valerate. Inefficient biodegradation due to a lack of proper environmental conditions and natural microbiota leads to microplastic accumulation and causes detrimental environmental impacts on most ecosystems.

This review summarizes recent advances in the biological upcycling of biodegradable plastics, such as PLA, PBAT, PBS, PCL, and PHAs, emphasizing the transition from simple environmental degradation to integrated carbon recovery platforms for manufacturing high-value products. We discuss enzymatic depolymerization strategies that generate defined biodegradable plastic monomers and compare these biocatalytic approaches with chemical depolymerization, highlighting the progress in enzyme discovery and protein engineering to expand substrate scope and catalytic efficiencies. We further evaluate pathway engineering and genetic rewiring, which enable native and engineered microorganisms, as monocultures or consortia, to bio-funnel carbon flux toward next-generation bioplastic precursors. We propose a unifying conceptual framework that integrates synthetic plastics into a biodegradable loop, enabling enzymatic deconstruction of heterogeneous plastic streams and metabolic upgrading into renewable precursors using a microbial bio-funneling approach. By combining these approaches with techno-economic assessments (TEA) and life-cycle analyses (LCA), engineered microbial systems have emerged as scalable platforms that can complement and outperform natural degradation pathways, thereby transforming plastic waste from an end-of-life burden into a regenerative carbon reservoir that moves toward true material circularity. The conceptual framework of microbial plastic upcycling and its integration with circular bioeconomy principles is illustrated in Figure 1.

## 2 Enzymatic degradation of biodegradable plastics

Enzymatic depolymerization is a central biological mechanism that enables the breakdown of biodegradable plastics into assimilable monomers, which can subsequently enter microbial metabolic pathways. Unlike purely chemical recycling approaches, enzyme-mediated degradation operates under mild conditions and offers substrate specificity that can be harnessed for selective polymer hydrolysis (Dey et al., 2025). However, the efficiency of enzymatic degradation varies substantially among biodegradable plastic classes because of differences in polymer chemistry, structural properties, and environmental conditions.

### 2.1 Factors affecting the enzymatic degradability of biodegradable plastics

Bioplastics are defined by two independent characteristics: feedstock origin (bio-based vs. fossil-derived) and end-of-life behavior (biodegradable vs. non-biodegradable). Several biodegradable polymers, such as PLA, PHAs, PBS, and starch-based blends, are produced from renewable biomass feedstocks that can naturally be degraded by enzymes. Certain biodegradable polymers, such as PBAT and PCL, are synthesized from fossil-derived monomers and remain enzymatically degradable because of their chemical structures (Gross and Kalra, 2002). Conversely, some bio-based polymers, including polyethylene (PE), polyethylene furanoate (PEF), polyethylene terephthalate (PET), and poly(trimethylene terephthalate) (PTT), are not biodegradable despite their renewable origin, because they are chemically identical to their petroleum-derived counterparts and therefore remain resistant to enzymatic degradation (Lackner et al., 2023). Thus, the designation “bioplastic” does not inherently imply enzymatic degradation.

The primary determinant of enzymatic degradation is the chemical structure of the polymer, particularly the type of bond in the backbone. Polymers containing hydrolysable functional groups, such as esters, amides, and glycosidic linkages, are generally susceptible to enzymatic cleavage because these bonds can be recognized and hydrolyzed by natural enzymes (Guebitz and Cavaco-Paulo, 2008). In contrast, polymers dominated by carbon–carbon (C-C) backbones exhibit a strong resistance to enzymatic attack because of the absence of chemically labile bonds (Jiao et al., 2025). In addition to polymer chemistry, several physicochemical properties significantly influence enzymatic degradability. Key structural parameters include polymer crystallinity, molecular weight, hydrophobicity, surface morphology, and the presence of additives, all of which affect enzyme adsorption and substrate accessibility (Zheng et al., 2025; Pérez-García et al., 2025). Highly crystalline regions often limit enzyme penetration, whereas amorphous regions are more readily hydrolyzed by enzymes than crystalline regions. Furthermore, environmental conditions, including temperature, pH, moisture availability, and microbial community composition, strongly influence enzymatic activity and degradation rates (Cai Z. et al., 2023). Together, these structural and environmental factors determine the enzymatic depolymerization efficiency and explain why multiple enzyme classes are often required to effectively degrade chemically diverse biodegradable plastics.

### 2.2 Major enzyme classes in biodegradable plastic degradation

**Figure 2:**
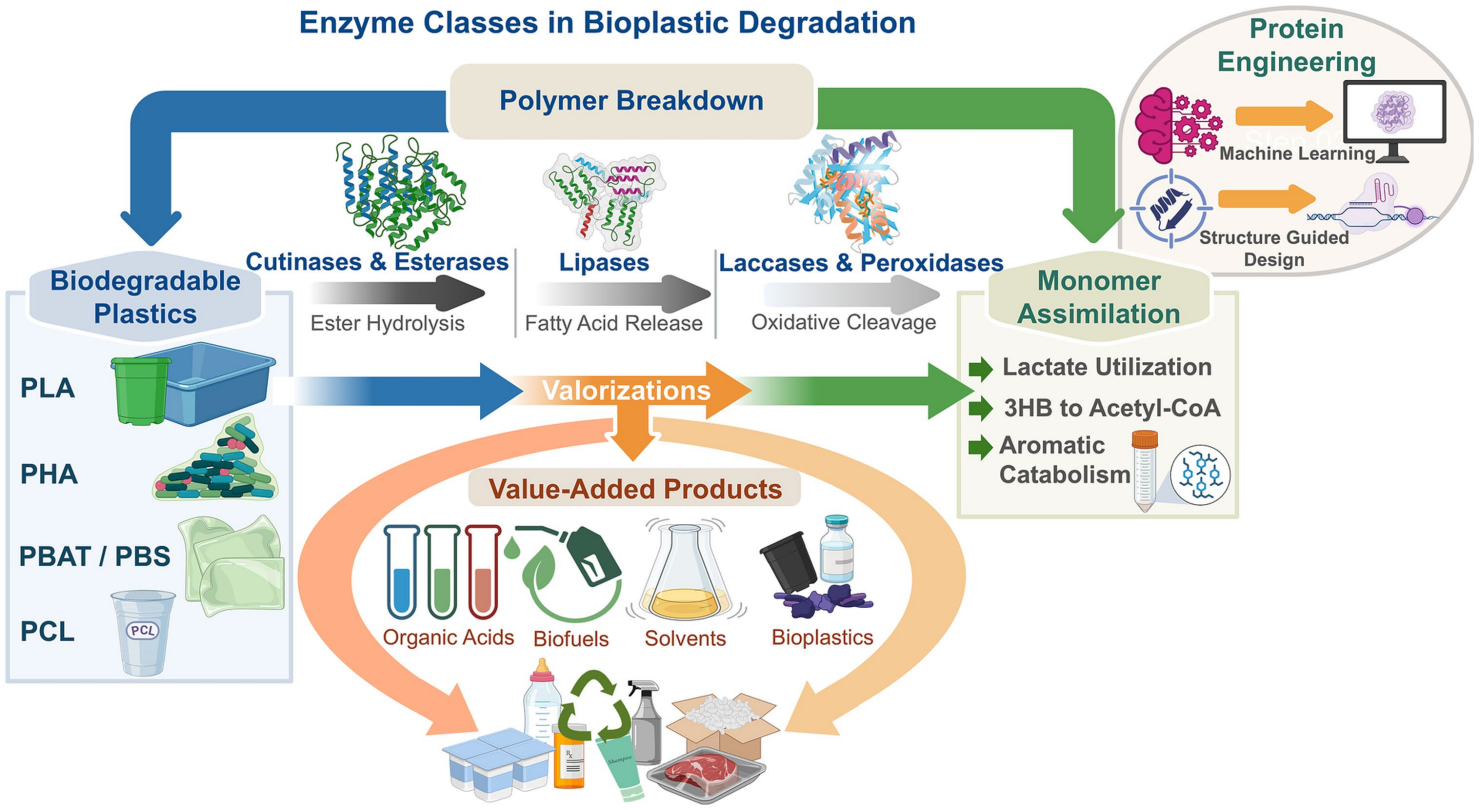
Enzyme classes and metabolic bio-funneling strategies in biodegradable plastic upcycling. Schematic overview of enzymatic depolymerization, monomer assimilation, protein engineering, and downstream valorization pathways of biodegradable polymers. Biodegradable plastics, including PLA, PHA, PBAT, polybutylene succinate (PBS), and PCL, are depolymerized primarily by hydrolases (cutinases, esterases, lipases) through ester bond hydrolysis and oxidoreductases (laccases and peroxidases), which facilitate oxidative cleavage and surface modification. The released intermediates, such as lactate, 3-hydroxybutyrate (3HB), and aromatic compounds, enter central metabolism via lactate utilization, β-oxidation/acetyl-CoA generation, and aromatic catabolic pathways. Engineered metabolic networks redirect these intermediates toward value-added products, including organic acids, biofuels, solvents, and next-generation bioplastics. Protein engineering approaches, such as machine learning-guided design and structure-based optimization, enhance enzyme stability, activity, and substrate specificity. Together, these integrated strategies enable the transformation of biodegradable plastic waste into renewable carbon feedstock for a circular bioeconomy.

Polymer-degrading enzymes, including those that act on plastics, can be classified into two major groups: extracellular and intracellular enzymes, which are broadly recognized for their depolymerase activity (Gu, 2003). During depolymerization, complex polymers are broken down into smaller chains or molecules, such as monomers, dimers, and oligomers. The major classes of enzymes involved in biodegradable plastic degradation are hydrolases (esterases, lipases, cutinases, and proteases), oxidoreductases (laccases and peroxidases), depolymerases (PETase, MHETase, and PHA depolymerases), carbohydrate-active enzymes (CAZymes), including amylases, cellulases, and hemicellulases, and accessory (auxiliary) enzymes, including oxidases, monooxygenases, and dehydrogenases, which act alongside the primary depolymerases. These enzymes facilitate degradation by modifying polymer surfaces, breaking down additives, or generating reactive intermediates that enhance accessibility for primary depolymerases (Pérez-García et al., 2025; Jiao et al., 2025; Lin et al., 2025). Figure 2 illustrates the integrated framework for enzyme-mediated polymer breakdown, monomer assimilation, protein engineering enhancement, and downstream valorization pathways in the upcycling of biodegradable plastics.

### 2.3 Protein/enzyme engineering advances

Protein engineering supports bioplastic upcycling with two distinct objectives: improving the depolymerases that break polymers into monomers and downstream metabolic enzymes that convert monomers into value-added products. It has improved the practical performance of enzymes used for the depolymerization of biodegradable plastics, particularly polyesters, such as PLA and PHAs. Directed evolution and structure-guided design have enhanced the catalytic activity, thermal stability, and substrate compatibility of cutinases, lipases, esterases, and PHA depolymerases that act on semicrystalline polymers. These modifications improve enzyme binding at the polymer surface, enable operation at elevated temperatures, and reduce product inhibition, collectively allowing for faster and more controlled depolymerization. Such improvements are especially important for polymers such as PLA and PBAT, where efficient enzymatic degradation depends on both thermal activation and appropriate active site geometry (Rahmati et al., 2024; Tournier et al., 2023; Crystal Thew et al., 2024; Bergeson et al., 2024).

The second key advancement is the development of enzymes that remain active under realistic processing conditions and in chemically heterogeneous waste streams (Jayasekara et al., 2023). Recent studies have shown that engineered hydrolases can function in high-solids systems, tolerate common plastic additives, and operate across wide pH and salinity ranges, extending their applicability beyond idealized laboratory buffers to composting, soil, and marine-like environments (Cui et al., 2024). Strategies such as the fusion of polymer-binding domains, surface modifications that limit aggregation, and improved secretion signals in microbial hosts have enhanced enzyme expression, stability, and reuse, thereby lowering overall enzyme requirements. Notably, enzyme-embedded bioplastics can be used for programmable degradation in the environment after application; however, maintaining material properties is challenging due to limited enzyme stability (Sun, 2025). Increasing attention has been paid to depolymerases tailored for mixed bioplastic streams, such as PLA/PBAT blends, where selective processing of chemically distinct ester linkages is essential for effective end-of-life treatment (Crystal Thew et al., 2024; Bergeson et al., 2024; Rezaei et al., 2024; Rahmati et al., 2024).

In contrast to the depolymerization-oriented engineering described above, protein engineering is increasingly being applied to metabolic enzymes that convert bioplastic-derived monomers into value-added products, directly linking degradation with valorization. Relieving feedback inhibition of polymer-hydrolyzing enzymes due to monomer accumulation is critical for sustaining throughput in consolidated single-pot depolymerization-fermentation systems. Engineered dehydrogenases, CoA-transferases, and polymerases can redirect lactic acid and hydroxyalkanoate monomers toward new biopolymers, specialty chemicals, and tailored PHA copolymers with enhanced material properties (Knott et al., 2020). Computational design and machine-learning-guided approaches accelerate the optimization of depolymerases and downstream metabolic enzymes, substantially shortening development timelines. Collectively, these advances have repositioned enzymes from passive degradation catalysts to programmable biocatalysts that support closed-loop recycling and bio-upcycling of biodegradable plastics (Joho et al., 2024; Tournier et al., 2023; Bergeson et al., 2024; Rezaei et al., 2024).

When viewed comparatively, rather than by study, the engineered depolymerase literature shows a consistent pattern of results. Reported gains in catalytic efficiency are the largest for low-crystallinity polyesters, engineered cutinases, and PETase variants assayed on amorphous films; the same enzymes show markedly reduced activity on semi-crystalline, additive-containing, or weathered materials. Thus, headline kinetic parameters tend to overstate the real-world performance (Thomsen et al., 2022; Kaabel et al., 2021). Thermostability improvements are industrially the most consequential gains because operation near the polymer glass-transition temperature increases chain mobility and accessibility. However, elevated-temperature operation also increases energy costs and must be justified against this trade-off (Thomsen et al., 2022). Substrate realism remains the weakest point: few studies have benchmarked engineered enzymes on genuine post-consumer feedstock or mixed polymer blends, crystallinity is frequently uncontrolled between studies, and scalability is rarely demonstrated beyond the bench-scale. Therefore, future enzyme-engineering work should be evaluated not only on k_cat_/K_M_ gains, but also on crystallinity tolerance, additive tolerance, performance on authentic waste, and reusability, which are the parameters that govern industrial feasibility.

**Figure 3:**
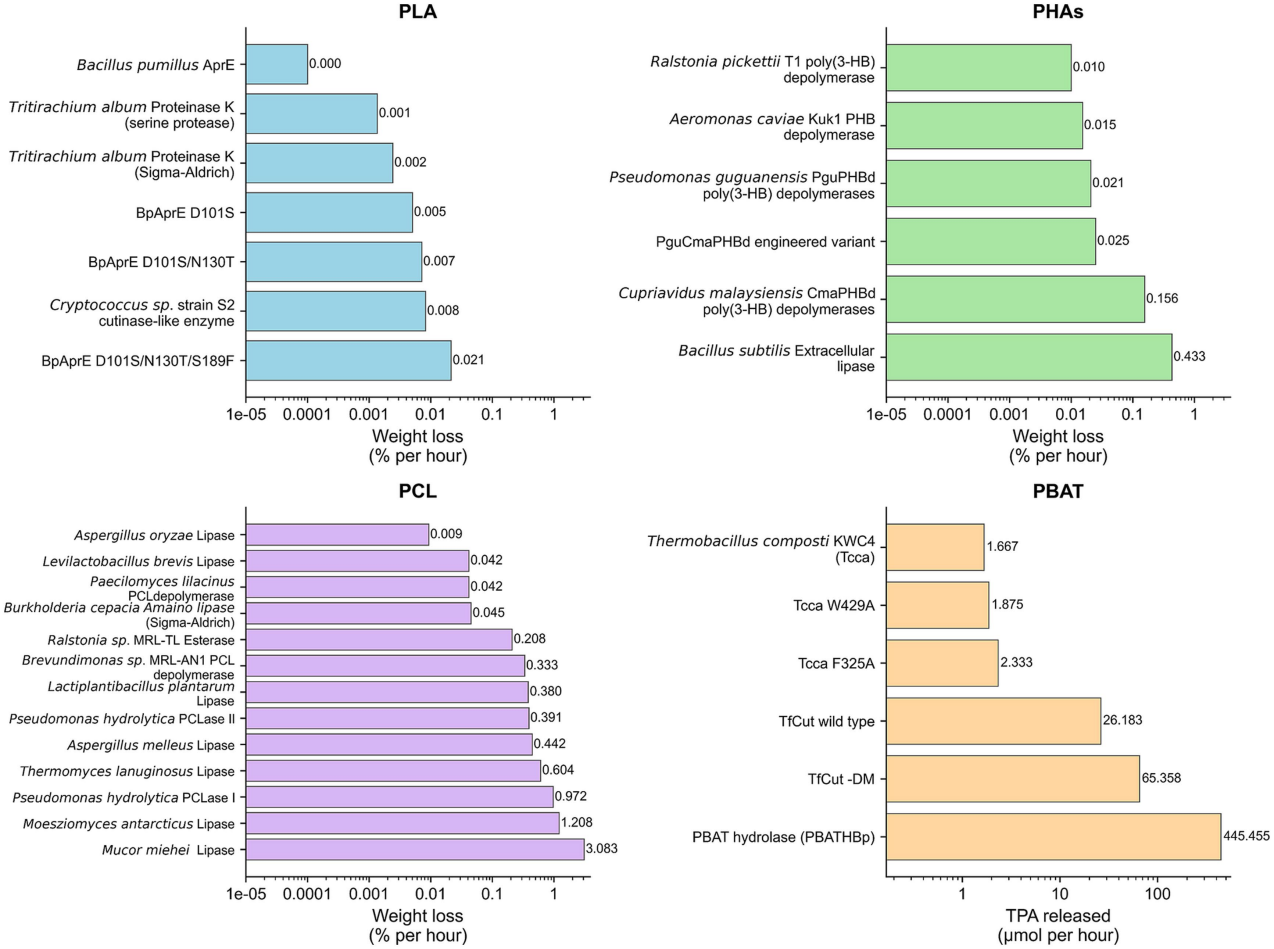
Comparative enzymatic depolymerization performance of major biodegradable plastics. Horizontal bar plots compare the reported depolymerization activities of representative enzymes acting on four biodegradable plastic substrates: polylactic acid (PLA), polyhydroxyalkanoates (PHAs), polycaprolactone (PCL), and polybutylene adipate-terephthalate (PBAT). For PLA, PHAs, and PCL, enzymatic activity was expressed as polymer weight loss (% per hour) derived from reported depolymerization assays. For PBAT, where studies typically report soluble product formation rather than direct polymer mass loss, activity is presented as the terephthalate (TPA) release rate (μmol h^−1^) following enzymatic hydrolysis.

The major enzyme classes involved in biodegradable plastic depolymerization, along with their kinetic properties, substrate specificity, reaction conditions, engineered variants, and downstream metabolic products, are summarized in Table 1. A comparative overview of the reported depolymerization activities across representative biodegradable polyester substrates is shown in Figure 3.

**Table 1:** Representative enzymes for the depolymerization of biodegradable plastics: catalytic properties, substrate specificity, engineered variants, and downstream metabolic fate.

Biodegradable plastic (source/type)	Depolymerizing enzyme (EC no.; source organism)	Enzyme class	Reported kinetic parameters	Substrate specificity	Optimal reaction conditions	Engineered variants	Downstream metabolic fate	References
*Polylactic acid (PLA)* Bio-based aliphatic polyester; biodegradable, typically under industrial composting	Proteinase K (EC 3.4.21.64; *Tritirachium album*)	Hydrolase	V_max_ = 0.03 mg min^−1^ K_M_ = 8 mg mL^−1^	Poly(L-lactic acid) (PLLA)	pH 8.6; 37 °C	n.r.	Lactate released; oxidized by lactate dehydrogenase/lactate oxidase to pyruvate; entry into central metabolism	Kawai et al. (2011), Hajighasemi et al. (2016), Cui et al. (2022), Shalem et al. (2024)
	Cutinase-like enzyme (CLE) (EC 3.1.1.74; *Cryptococcus* sp. S-2)	Hydrolase	V_max_ = 0.04–0.09 mg min^−1^ K_M_ = 10 mg mL^−1^	PLLA and poly(D-lactic acid) (PDLA)	pH 7.0; 37 °C	n.r.		
	Lipase (Novozym 42,044) (EC 3.1.1.3; commercial preparation)	Hydrolase	V_max_ = 0.02 mg min^−1^ K_M_ = 10 mg mL^−1^	PLLA and PDLA	pH 7.0; 30 °C	n.r.		
	Esterase ABO2449 (EC 3.1.1.–; *Alcanivorax borkumensis*)	Hydrolase	k_cat_ = 195.1 ± 4.4 s^−1^ K_M_ = 0.24 ± 0.02 mM k_cat_/K_M_ = 0.81 s^−1^ μM^−1^	D, L-PLA; α-naphthyl acetate; PCL and other polyesters; soluble α-naphthyl and *p*-nitrophenyl monoesters	pH 9.5–10.0; 30–37 °C (thermostability T_m_ 32.3 ± 0.5 °C)	M144A, L163A, M183A, F219A, P302R; all variants showed lower K_M_ than wild type		
	Esterase RPA1511 (EC 3.1.1.–; *Rhodopseudomonas palustris*)	Hydrolase	n.r.	D, L-PLA	pH 9.5–10.0; 55–60 °C (thermostability T_m_ 70.8 ± 0.5 °C)	RPA1511-V202A; markedly higher PLA-hydrolyzing activity than wild-type RPA1511		
*Polyhydroxyalkanoates (PHAs; e.g., PHB, PHBV)* Bio-based microbial storage polyesters; biodegradable	Extracellular PHA depolymerase (e-PHA) [EC 3.1.1.75; *Aeromonas caviae* Kuk1-(34)]	Depolymerase	V_max_ = 1.89 U mL^−1^ K_M_ = 0.77 mg mL^−1^	Extracellular paracrystalline PHA granules; short-chain-length PHA	pH 8.0 (range 7.0–9.0); 35 °C (range 35–45 °C)	n.r.	3-Hydroxybutyrate released; oxidized by 3-hydroxybutyrate dehydrogenase and routed via CoA-transferase to acetoacetyl-CoA and acetyl-CoA, entering the TCA/glyoxylate cycles	Amir et al. (2024), Amir et al. (2022), Knoll et al. (2009)
	Intracellular PHB depolymerase PhaZa1 (EC 3.1.1.75; *Cupriavidus necator*; also, PhaZ1 of *Bacillus megaterium*)	Depolymerase	n.r.	Native intracellular PHA granules	pH 8.0–8.5; 40–45 °C	n.r.		
	Extracellular MCL-PHA depolymerase (EC 3.1.1.76; *Thermus thermophilus* HB8)	Depolymerase	K_M_ = 53.2 mM (*p*-nitrophenyl octanoate, pNPO)	Medium-chain-length PHAs, e.g., poly(3-hydroxyhexanoate), poly(3-hydroxyoctanoate)	pH 7.5–9.0; 70 °C (pNPO hydrolysis)	n.r.		
*Polycaprolactone (PCL)* Fossil-derived aliphatic polyester; biodegradable	Lipase (EC 3.1.1.3; *Penicillium fellutanum*)	Hydrolase	V_max_ = 83.3 μmol mL^−1^ min^−1^ K_M_ = 0.75 mM (*p*-nitrophenyl palmitate, pNPP)	Mono-, di-, and triglycerides; PCL	pH 8.5; 45 °C	n.r.	6-Hydroxyhexanoate released; processed via β-oxidation-type dehydrogenase steps into the acetyl-CoA pool	Amin et al. (2022), Ravi et al. (2025), Almeida et al. (2019), Li et al. (2022)
	PCLase I (cutinase) and PCLase II (esterase) (EC 3.1.1.74/3.1.1.–; *Pseudomonas hydrolytica* DSWY01)	Hydrolase	n.r.	PCL, PBS, *p*-nitrophenyl esters, tributyrin, olive oil; PCLase I also degrades crude cutin, PCLase II also degrades PHB	PCLase I: pH 9.0, 50 °C PCLase II: pH 10.0, 40 °C	n.r.		
*Poly(butylene adipate-co-terephthalate) (PBAT)* Aliphatic–aromatic copolyester, partly fossil-derived; biodegradable	Esterase PpEst (EC 3.1.1.–; *Pseudomonas pseudoalcaligenes*)	Hydrolase	k_cat_ = 1.98 s^−1^ K_M_ = 4.85 mM	Milled PBAT, PLA, and PET films; BABuTABuBA model substrate	pH 8.0; 65 °C	n.r.	Aromatic moiety routed through protocatechuate catabolism; adipate and 1,4-butanediol are oxidized into central metabolism	Wallace et al. (2017), Wu et al. (2023), Yang et al. (2023)
	Serine hydrolase TfH (EC 3.1.1.74; *Thermobifida fusca*)	Hydrolase	k_cat_ = 220 s^−1^ K_M_ = 0.62 mM	PBAT	pH 8.0; 50 °C	n.r.		
	Lipase PfL1 (EC 3.1.1.3; *Pelosinus fermentans*)	Hydrolase	k_cat_ = 5 s^−1^ K_M_ = 6.57 mM	PBAT	n.r.	n.r.		
	Esterases Cbotu_EstA and Cbotu_EstB (EC 3.1.1.–; *Clostridium botulinum*)	Hydrolase	EstA: k_cat_ = 71.9 s^−1^, K_M_ = 1.95 mM EstB: k_cat_ = 5.84 s^−1^, K_M_ = 1.30 mM	Soluble esterase substrates *p*-nitrophenyl acetate (pNPA) and *p*-nitrophenyl butyrate (pNPB)	EstA: pH 7.0–8.0, 60 °C (active 20–70 °C) EstB: 40 °C (active 20–60 °C)	n.r.		
	Cutinase Thc_Cut1 (EC 3.1.1.74; *Thermobifida cellulosilytica*)	Hydrolase	k_cat_ = 325 s^−1^ K_M_ = 0.8 mM	PBAT	n.r.	n.r.		
	Cutinase TfCut (EC 3.1.1.74; *Thermobifida fusca*)	Hydrolase	n.r.	Terephthalate-containing moiety of PBAT	n.r.	TfCut-DM (double mutant); faster hydrolysis than wild-type TfCut; complete PBAT decomposition in 36 h vs. 48 h for wild type		
*Poly(butylene succinate) (PBS) / PBSA* Aliphatic polyester, bio-based or fossil-derived; biodegradable	Lipases and cutinase-like esterases (EC 3.1.1.3/3.1.1.74; e.g., *Cryptococcus* sp.)	Hydrolase	n.r.	PBS, PBSA	n.r.	n.r.	Succinate and 1,4-butanediol released; diol dehydrogenases oxidize diols into TCA-cycle intermediates	Wcisłek et al. (2018), Thirunavukarasu et al. (2016)
*Poly(ethylene 2,5-furanoate) (PEF)* Bio-based PET analog; degradability depends on enzyme and crystallinity	PET hydrolases and cutinases/esterases (EC 3.1.1.101/3.1.1.74; e.g., LCC, PETase variants)	Hydrolase	n.r.	PEF; PET	n.r.	Engineered thermostable PET-hydrolase variants (e.g., LCC_ICCG_, FAST-PETase) applied to furanoate polyesters	2,5-Furandicarboxylic acid (FDCA) and ethylene glycol released; FDCA assimilation or recovery for repolymerization; EG oxidized via dehydrogenases	Kawai et al. (2022)
*Starch- and cellulose-based blends* Bio-based polysaccharide blends; biodegradable	Carbohydrate-active enzymes (CAZymes): amylases, cellulases, hemicellulases (EC 3.2.1.1/3.2.1.4/3.2.1.–)	CAZyme	n.r.	Starch (amylases); cellulose and plant fibers (cellulases, hemicellulases)	n.r.	n.r.	Saccharification to mono- and oligosaccharides; sugars enter glycolysis	Yao et al. (2024), Erdal and Hakkarainen (2022)
*Poly(ester amide)s / polyamide-4 (PA4) and related nylons* Amide-containing polymers; selected grades (e.g., PA4) bio-based and biodegradable	Nylonases NylA, NylB, NylC (EC 3.5.2.12/3.5.1.46/3.5.1.117; *Paenarthrobacter ureafaciens*, plasmid pOAD2)	Accessory enzyme	n.r.	NylA: cyclic Ahx-dimer → linear Ahx-dimer; NylB: linear Ahx-oligomer exohydrolase; NylC: cyclic and linear Ahx-oligomer endohydrolase	n.r.	n.r.	Aminohexanoate units released; deaminases and transaminases route carbon and nitrogen into central metabolism	Takehara et al. (2017) and de Witt et al. (2024)

Kinetic parameters are reported as provided in the cited sources; symbols follow standard conventions (K_M_, Michaelis constant; k_cat_, turnover number; V_max_, maximum reaction velocity). The temperatures are given in degrees Celsius (°C), and the reaction pH is a unitless value. Mutant variants follow the single-letter convention (wild-type residue, position, substituted residue); n.r., not reported.

Several enzyme classes, including proteases and engineered subtilisin variants, have been reported to depolymerize PLA. Native proteases, such as Proteinase K from *Tritirachium album* and commercial Proteinase K preparations, exhibit measurable PLA hydrolysis activity, reflecting the ability of serine proteases to cleave ester linkages in aliphatic polyesters. Engineered variants of BpAprE, a subtilisin-like protease derived from *Bacillus pumilus*, including D101S, D101S/N130T, and D101S/N130T/S189F, exhibited progressively improved catalytic performance, illustrating how targeted mutations can enhance polymer-enzyme interactions and substrate accessibility. Additionally, a cutinase-like enzyme from *Cryptococcus* sp. strain S-2 demonstrated effective PLA hydrolysis, which is consistent with the well-established role of fungal and yeast cutinases in the degradation of polyesters. These results collectively indicate that both protease-derived and cutinase-like enzymes can contribute to PLA depolymerization, with engineered variants generally outperforming native enzymes owing to their improved catalytic efficiency and substrate affinity (Shrestha et al., 2025; Cui et al., 2022; Cannon and Reynolds, 2023; Kawai et al., 2011).

For PHA degradation, the enzymes represented are primarily PHB depolymerases, which have evolved naturally to hydrolyze polyhydroxybutyrate and related polyhydroxyalkanoates. Several bacterial depolymerases exhibit measurable activity toward PHB polymers, including PguPHBd from *Pseudomonas guguanensis* and Kuk1 PHB depolymerase from *Aeromonas caviae*, both of which catalyze the cleavage of ester bonds within poly(3-hydroxybutyrate) (Kawai et al., 2011). An engineered PguCmaPHBd variant further demonstrated that protein engineering can enhance depolymerase activity relative to that of native enzymes. Among the enzymes compared, CmaPHBd from *Cupriavidus malaysiensis* exhibited relatively high hydrolytic activity, reflecting the evolutionary specialization of *Cupriavidus* species in PHA metabolism. In addition to canonical depolymerases, extracellular lipases from *Bacillus subtilis* exhibit measurable PHA depolymerization activity, suggesting that lipases may function as auxiliary polyester-degrading enzymes under specific conditions. Collectively, these results highlight the diversity of bacterial enzymes capable of degrading PHAs and emphasize the importance of depolymerase specialization for efficient polymer hydrolysis (Amir et al., 2024; Koike et al., 2024; Bodourian et al., 2026; Palanisamy et al., 2016).

The enzyme landscape for PCL degradation is considerably broader, indicating higher susceptibility to hydrolysis. Multiple fungal and bacterial enzymes exhibit measurable activity toward PCL substrates. Lipases from *Aspergillus oryzae*, *Levilactobacillus brevis*, and *Paecilomyces lilacinus* exhibited moderate hydrolysis rates, indicating that both fungal and bacterial lipases can effectively cleave PCL ester bonds. Additional enzymes, such as Amano lipase from *Burkholderia cepacia* and MRL-TL esterase from *Ralstonia* sp., further demonstrate the versatility of microbial esterases in the degradation of polyesters. Enzymes from *Brevundimonas* sp., *Lactiplantibacillus plantarum*, and *Pseudomonas hydrolytica* exhibit enhanced catalytic activity, particularly variants such as PCLase I and PCLase II, which are specialized in PCL hydrolysis. Among the most active enzymes in this dataset are lipases derived from *Aspergillus melleus*, *Thermomyces lanuginosus*, *Moesziomyces antarcticus*, and *Mucor miehei*, which have been widely studied for their robust polyester-degrading activities. The broad range of enzymes capable of degrading PCL underscores the relative susceptibility of this polymer to enzymatic hydrolysis and highlights the important role of lipases as versatile polyester-degrading catalysts (Dias et al., 2022; Shah et al., 2015; Zhang and Qiu, 2016; Li et al., 2022; Khan et al., 2017; Federle et al., 2002; Yang et al., 2015; Antipova et al., 2018).

In contrast to the weight loss measurements used for PLA, PHA, and PCL, depolymerization studies for PBAT commonly quantify soluble product formation, particularly the release of terephthalate (TPA) following enzymatic hydrolysis. Accordingly, the PBAT panel reports enzymatic activity as the TPA release rate (μmol h^−1^) rather than polymer mass loss. Among the enzymes compared, TccA from *Thermobacillus composti* and its engineered variants, TccA W429A and TccA F325A, demonstrated measurable depolymerization activity toward PBAT. More substantial catalytic activity was observed for TfCut, a cutinase originally identified from *Thermobifida fusca*, particularly for its engineered TfCut-DM mutant, which was optimized for enhanced polyester hydrolysis. The highest activity in this comparison was observed for PBAT hydrolase (PBATHBp), which released substantially higher amounts of terephthalate, illustrating the potential of specialized polyester hydrolases for efficient PBAT depolymerization (Soulenthone et al., 2021; Kanwal et al., 2022; Wu et al., 2023; Muroi et al., 2017).

This comparative analysis highlighted several key trends in enzymatic polyester degradation. First, polymer susceptibility strongly influences the diversity of effective enzymes. PCL is degradable by a wide range of lipases and esterases, whereas PHA degradation primarily relies on specialized PHB depolymerases. Second, enzyme engineering is critical for enhancing catalytic performance, as demonstrated by engineered variants of BpAprE, PguPHBd, TfCut, and TccA. Finally, activity measurements vary across substrates, with weight-loss metrics commonly used for aliphatic polyesters such as PLA, PHA, and PCL, whereas aromatic–aliphatic copolymers such as PBAT are typically assessed through soluble product formation. These comparisons illustrate how enzyme discovery and engineering can provide a catalytic foundation for efficient biodegradable plastic depolymerization and subsequent microbial recycling/upcycling pathways.

## 3 Pathway and strain engineering

Microorganisms function as cellular factories that both produce and degrade biodegradable plastics. Although wild-type microbes naturally secrete depolymerizing enzymes and produce bioplastic monomers, pathway and strain engineering are essential to enhance monomer yields and accelerate the degradation rates (de Souza and Gupta, 2024; Nduko and Taguchi, 2020). Biodegradable plastic deconstruction generates a diverse range of monomers and oligomers depending on the polymer type, enzymatic activity, and environmental conditions. Numerous bacterial and fungal genera possess the inherent capacity to degrade plastics in natural settings. However, released intermediates may exert ecotoxic effects if they are not metabolized further (Hu et al., 2023). Therefore, engineered microbial systems capable of assimilating, detoxifying, and valorizing these intermediates are critical for preventing secondary pollution and enabling the sustainable upcycling of biodegradable plastics.

### 3.1 Engineered microbes for efficient biodegradation of plastics

Bioengineered bacteria, fungi, and microalgae have transformed the depolymerization of biodegradable plastics by serving as robust biocatalysts developed using advanced synthetic biology tools. Engineering enzymes for improved activity, stability, and wider substrate range involves key approaches such as cell surface display of plastic-degrading enzymes, rewiring of metabolic pathways using genetic tools such as CRISPR-Cas9, and development of microbial consortia (Dissanayake and Jayakody, 2021; Wang and Shi, 2025).

Recombinant expression of a fungal cutinase-like enzyme (CLE1) in yeast (*Saccharomyces cerevisiae*) has enabled the production of high-titer crude PLA hydrolases for efficient PLA degradation (Myburgh et al., 2023). In addition, culture condition optimization offers an opportunity to use yeast and bacteria for PLA depolymerization (Boonluksiri et al., 2021; Shalem et al., 2024). PHB biodegradation has been enhanced through engineering of PHB-degrading PhaZ enzymes, upregulation of PhaZ gene expression, optimized conditions, and use of mixed microbial consortia (Park et al., 2022; Chae and Lee, 2025). Efficient biodegradation of PCL has been achieved by optimizing enzymes such as cutinases from *Mycobacterium marinum* (MmCut3), recombinant production of PCL depolymerases in hosts such as *E. coli*, immobilization of enzymes such as lipases in the PCL matrix, and metabolic pathway engineering in strains including *Acinetobacter* (Khan et al., 2019; Ravi et al., 2025; Oh J. Y. et al., 2022; Sankhla et al., 2020).

Higher degradation of PBS has been demonstrated using artificial microbial consortia, such as *Bacillus* JY35 and NR4 strains, as well as heterologous expression of PBS-degrading enzyme genes in *E. coli*, optimization of fungal cutinases, such as AaCut10 (Kimura et al., 2023; Shin et al., 2024; Hu et al., 2024). Similarly, PBAT biodegradation can be enhanced by rational enzyme engineering of cutinase from *Thermobifida fusca*, engineered consortia, and fine-tuning of biodegradation conditions (Fernandes et al., 2025; Yang et al., 2023; Wang Y. et al., 2025a).

### 3.2 Engineered microorganisms to assimilate plastic-derived monomers

In addition to the capabilities of naturally occurring microbes to assimilate plastic-derived monomers, the introduction of heterologous pathways or the overexpression of native pathway genes has been shown to further enhance the efficient assimilation of biodegradable plastic monomers. For instance, certain bacteria have an inherent capability to consume lactic acid and convert it into pyruvate, which then enters the TCA cycle. Lactate utilization has been enabled in several genera of microorganisms for several applications, such as biopolymer production, photosynthetic carbon capture, cancer therapy, biochemical production, and biosensor development (Wang Y. et al., 2025a; Zúñiga et al., 2021; Jiang et al., 2014). In these microbes, lactate metabolism has been enabled or improved by introducing lactate-metabolizing genes, such as lactate dehydrogenase and lactate permease, into hosts such as yeast and cyanobacteria, optimizing promoter systems, as in the case of synthetic promoters specific for challenging conditions, and knocking out/knocking down competing pathway genes (Kato et al., 2023; Perveen et al., 2025; Matanović et al., 2025).

Several engineering approaches, primarily using *E. coli* and *P. putida* strains, have been developed to enable TPA metabolism by heterologously expressing TPA transporters and catabolic genes (Dissanayake and Jayakody, 2021). Researchers have also developed microbial consortia, or single strains, that efficiently degrade plastics, utilize TPA, and funnel into sustainable products while overcoming toxicity issues (Bao et al., 2023; Oh et al., 2024; Li Y. et al., 2024; Dissanayake et al., 2026). Common microbial chassis of ethylene glycol utilization includes *E. coli* and *P. putida* strains, where metabolism is enhanced by expressing the *gcl* operon or overexpressing genes such as *gcl*, *hyi*, *glxR*, and *glxK* for pathway enablement or by knocking out competing genes to redirect flux (Franden et al., 2018; Luo et al., 2023). The engineering approaches in these strains are compatible with the use of monomer(s) derived from bioplastics, such as PBAT.

TPS and cellulose-based plastics-derived glucose/cellobiose assimilation can be enhanced by adapting engineering strategies used for efficient metabolism, such as introducing missing pathway genes, including transporter and phosphorylation genes, overcoming glucose repression, and developing synthetic consortia (Choi et al., 2014; Lin et al., 2017; Seong et al., 2020). This improved glucose utilization has been demonstrated in a range of microbial hosts, including *E. coli*, *Shewanella* sp., *Methanosarcina acetivorans*, *Saccharomyces cerevisiae*, *Clostridium acetobutylicum*, and *Lactobacillus* sp. In addition, advanced bio-hybrid systems have been developed by integrating glucose-utilizing archaea with nanoparticles for direct glucose conversion (Ma et al., 2024).

Microorganisms can assimilate biodegradable plastics, facilitate their end-of-life, and minimize environmental pollution. This microbial ability is crucial for valorizing the untapped organic carbon in plastic waste, as it enables the conversion of plastic-derived monomers into useful biomass, energy, or valuable chemicals. This contributes to a circular economy in which plastic-derived carbon re-enters production as a feedstock. The microorganisms involved in the depolymerization and assimilation of biodegradable plastics, along with their corresponding monomers and metabolic products, are summarized in Table 2.

**Table 2:** Microbial depolymerization and assimilation of biodegradable plastics: representative degrading and assimilating microorganisms, released monomers, and resulting metabolic products.

Biodegradable polymer	Representative microbial degraders	Monomer(s) released on depolymerization	Representative microbial assimilators	Representative metabolic product(s)	References
PLA (polylactic acid)	*Bacillus brevis*, *B. licheniformis*, *B. smithii*, *B. amyloliquefaciens*, *Amycolatopsis orientalis*, *A. mediterranei*, *Streptomyces viridosporus*, *S. thermonitrificans*, *Pseudozyma antarctica*, *Aspergillus fumigatus*, *A. flavus*, *Penicillium chrysogenum*, *P. simplicissimum*, *Rhizopus oryzae*, *Geobacillus thermoleovorans*, *Caldicellulosiruptor bescii*, *Vibrio alginolyticus*, *V. coralliilyticus*, *Pseudoalteromonas* spp.	Lactic acid	*Mycobacterium tuberculosis*, *Acinetobacter* sp. strain WLIS, *Lactobacillus plantarum*, *L. casei*, *Aerococcus viridans*	Pyruvate	Torres et al. (1996), Pranamuda et al. (1997), Nakajima-Kambe et al. (2009), Karamanlioglu et al. (2014), Sankhla et al. (2020) and Wang et al. (2023)
		Lactic acid	*Propionibacterium freudenreichii*, *Veillonella parvula*, *Megasphaera elsdenii*, *Clostridium homopropionicum*, *C. neopropionicum*, *Anaerotignum propionicum*, *A. neopropionicum*, *Pelobacter propionicus*, *Phascolarctobacterium succinatutens*, *Dialister* spp.	Propionate, acetate	
		Lactic acid	*Anaerobutyricum soehngenii*, *Coprococcus catus*, *Anaerostipes caccae*, *Eubacterium hallii*, *Faecalibacterium prausnitzii*, *Roseburia intestinalis*, *Clostridium butyricum*, *Megasphaera indica*	Butyrate	
		Lactic acid	*Acetobacterium woodii*; *Shewanella oneidensis* MR-1; *Megasphaera elsdenii*	Acetate (*A. woodii*); acetate and pyruvate (*S. oneidensis*); acetate, propionate and butyrate (*M. elsdenii*)	
PHB (polyhydroxybutyrate)	*Pseudomonas lemoignei*, *P. stutzeri*, *P. putida*, *Streptomyces exfoliatus*, *S. albus*, *S. lividans*, *Bacillus megaterium*, *B. subtilis*, *B. cereus*, *Cupriavidus necator*, *C. taiwanensis*, *Alcaligenes faecalis*, *Comamonas testosteroni*, *C. acidovorans*, *Nocardia salmonicolor*, *N. corallina*, *Rhizobium meliloti*, *R. leguminosarum*, *Vibrio* spp., *Shewanella* spp., *Pseudoalteromonas* spp.	3-Hydroxybutyric acid; 3-hydroxyvaleric acid	*Cupriavidus necator*, *Bacillus* spp., *Pseudomonas* sp., *Enterobacter* sp., *Rhodospirillum rubrum*, *Rhodococcus* sp., *Caldimonas taiwanensis*, *Paracoccus denitrificans*, *Haloferax mediterranei*	Acetyl-CoA and ATP via 3-hydroxybutyrate dehydrogenase; re-polymerization to PHB and PHBV	Schöber et al. (2000), Jendrossek and Handrick (2002), Colak and Güner (2004), Calabia and Tokiwa (2006), Anjulal et al. (2024) and Huang et al. (2025)
PHBV (poly(3-hydroxybutyrate-co-3-hydroxyvalerate))	*Pseudomonas lemoignei*, *P. putida*, *Streptomyces exfoliatus*, *S. albus*, *Bacillus megaterium*, *B. cereus*, *Cupriavidus necator*, *Alcaligenes faecalis*, *Comamonas testosteroni*, *Acinetobacter calcoaceticus*, *Nocardia salmonicolor*, *Rhizobium meliloti*, *Vibrio* sp., *Shewanella* sp., *Pseudoalteromonas* spp.	3-Hydroxybutyric acid; 3-hydroxyvaleric acid	See PHB assimilators; 3-hydroxyvalerate additionally enters propionyl-CoA metabolism	Acetyl-CoA and propionyl-CoA; re-polymerization to PHBV with tunable 3 HV content	Aneja and Charles (1999), Sudesh et al. (2000), Bose et al. (2023) and Liu et al. (2024)
PCL (polycaprolactone)	*Fusarium solani*, *Aspergillus flavus*, *Penicillium* spp., *Pseudomonas fluorescens*, *Streptomyces roseolus*, *Streptomyces* sp., *Bacillus subtilis*, *B. pumilus*, *Acinetobacter* sp., *Thermobifida fusca*, *Pseudoalteromonas* spp., *Vibrio* spp.	6-Hydroxyhexanoic acid (from ε-caprolactone)	*Pseudomonas pseudoalcaligenes*, *P. hydrolytica*, *Alcaligenes faecalis*, *Streptomyces* spp., *Rhodococcus* spp., *Brevibacterium epidermidis* BS3, *Acinetobacter* strain SE19	Acetyl-CoA via β-oxidation-type dehydrogenase steps	Doi et al. (1992), Nakayama et al. (2019), Esikova et al. (2023) and Krumov et al. (2025)
PBS (poly(butylene succinate))	*Pseudomonas putida*, *P. alcaligenes*, *P. mendocina*, *Aspergillus versicolor*, *Fusarium solani*, *Paraphoma chrysanthemicola*, *Humicola insolens*, *Bacillus licheniformis*, *B. pumilus*, *B. subtilis*, *B. cereus*, *Amycolatopsis* spp., *Acinetobacter baumannii*, *Marinibacterium* sp., *Sulfitobacter* sp., *Rhodococcus* spp.	Succinic acid	*Phascolarctobacterium faecium*, *Propionigenium modestum*, *P. maris*, *Rhodovulum sulfidophilum*	Propionate, acetate, CO₂; polyhydroxyalkanoates	Aliotta et al. (2022), Kim J. et al. (2023), Hu et al. (2024) and Jang et al. (2025)
		1,4-Butanediol	*Pseudomonas putida* KT2440, *Ustilago trichophora*, *Corynebacterium glutamicum*	Succinate; 4-hydroxybutyrate	
PBSA (poly(butylene succinate-co-adipate))	*Bacillus* sp. (JY35, NR4), *B. pumilus*, *B. stearothermophilus*, *B. subtilis*, *Laceyella sacchari*, *Terribacillus goriensis*, *Thermomonospora fusca*, *Thermobifida alba*, *Roseateles depolymerans*, *Halopseudomonas* sp. MFKK-1, *Vibrio ruber*, *V. rhizosphaerae*, *V. spartinae*, *Fusarium solani*, *Aspergillus versicolor*, *Bionectria ochroleuca*, *Penicillium* sp.	Succinic acid; 1,4-butanediol	See PBS assimilators	Propionate, acetate, CO₂; polyhydroxyalkanoates	Zhao et al. (2005), Kim et al. (2025), Kimura et al. (2023) and Soulenthone et al. (2025)
		Adipic acid	*Oceanihabitans sediminis*, *Pseudomonas putida* KT2440, *Acinetobacter baylyi* ADP1, *Cupriavidus necator* H16	Acetyl-CoA; polyhydroxyalkanoates; TCA-cycle intermediates	
PBAT (poly(butylene adipate-co-terephthalate))	*Bacillus amyloliquefaciens*, *B. pumilus*, *B. subtilis*, *Clostridium hathewayi*, *Escherichia coli*, *Leptothrix* sp. TB-71, *Peribacillus frigoritolerans* S2313, *Propionispora hippei*, *Rhodococcus* sp. NKCM2511, *Roseateles depolymerans* TB-87, *Roseibium aggregatum* ZY-1, and *Stenotrophomonas* sp. YCJ1, *Thermobispora bispora*, *Thermomonospora fusca*, *Alternaria alternata* FB1, *Aspergillus* spp., *Cladosporium* spp., *Cryptococcus* sp. MTCC 5455, *Purpureocillium lilacinum*, *Trichoderma harzianum*	Adipic acid; 1,4-butanediol	See PBSA adipate assimilators	Acetyl-CoA; TCA-cycle intermediates	Yoshida et al. (2016), Fei et al. (2024), Wang et al. (2024), Fernandes et al. (2025) and Molpeceres-García et al. (2025)
		Terephthalic acid	*Comamonas testosteroni*, *Ideonella sakaiensis* 201-F6, *Rhodococcus pyridinivorans* P23, *Pseudomonas chengduensis*, *P. umsongensis* GO16, *Acinetobacter* spp., *Achromobacter* spp., *Arthrobacter* spp., *Bacillus* spp., *Stenotrophomonas* spp., Var*iovorax* spp., *Streptomyces* spp.	Protocatechuate and catechol; cis,cis-muconate	
TPS (thermoplastic starch)	*Bacillus amyloliquefaciens*, *B. subtilis*, *B. licheniformis*, *B. stearothermophilus*, *B. megaterium*, *B. circulans*, *Klebsiella pneumoniae*, *Micromonospora* sp., *Nocardia* sp., *Streptomyces* sp., *Aspergillus oryzae*, *A. niger*, *A. awamori*, *Trichoderma* sp., *Clonostachys rosea*, *Gliocladium viride*, *Mortierella subtilissima*	Glucose; maltodextrins	*Escherichia coli*, *Saccharomyces cerevisiae*, *Lactobacillus plantarum*, *Bacillus subtilis*, *Clostridium acetobutylicum*, *Aspergillus niger*, *Pseudomonas aeruginosa*, *Clostridium thermocellum*, *Zymomonas mobilis*, *Enterococcus faecalis*	CO₂, lactate, acetate, ethanol, succinate, formate, acetoin, butanol, citric acid	Schlemmer et al. (2009), Canché-Escamilla et al. (2011), Polman et al. (2021), Syranidou et al. (2024), Erdal and Hakkarainen (2022) and Weimer (2022)
Cellulose-based plastics (e.g., cellulose acetate)	*Bacillus cereus*, *Pseudomonas putida*, *Pleurotus ostreatus*, *Lentinus lepideus*	Cellobiose	*Clostridium thermocellum*, *Ruminococcus albus*, *Saccharophagus degradans*, *Victivallis vadensis*, *Bacteroides* spp., *Cellvibrio gilvus*, *Saccharomyces cerevisiae*	Acetate, ethanol, lactate, H₂, CO₂, propionate, butyrate; polyhydroxyalkanoates; biomass	Erdal and Hakkarainen (2022), Weimer (2022) and Pulyala et al. (2025)
		Acetic acid	*Escherichia coli*, *Pseudomonas putida*, *Cupriavidus necator* H16, *Saccharomyces cerevisiae*, *Yarrowia lipolytica*, *Clostridium ljungdahlii*, *Methanosarcina acetivorans*, *Acetobacter aceti*	Biomass, CO₂, ethanol, methane; polyhydroxyalkanoates; lipids/triacylglycerols	
PEF (poly(ethylene 2,5-furanoate))	*Cryptococcus* sp., *Thielavia terrestris*, *Humicola insolens*, *Aspergillus* spp., *Fusarium* spp., *Penicillium* sp., *Ideonella sakaiensis*, *Pseudomonas* spp., *Rhodococcus* spp., *Bacillus* spp., *Arthrobacter* spp., *Comamonas testosteroni*, *Enterobacter* spp., *Serratia marcescens*	2,5-Furandicarboxylic acid (FDCA); ethylene glycol	*Pseudomonas putida*, *Paracoccus denitrificans*, *Rhodococcus jostii*, *Cupriavidus basilensis*, *Comamonas testosteroni*, *Acetobacterium woodii*, *Halomonas elongata*	FDCA: TCA cycle and ring-cleavage intermediates, acetyl-CoA derivatives. Ethylene glycol: glycolaldehyde, glycolate, glyoxylate, pyruvate	Cai Z. et al. (2023), Dolz et al. (2024), Wróbel et al. (2023), Balola et al. (2024) and Liu et al. (2025)

## 4 Tailoring microbial metabolism to upcycle biodegradable plastics-derived compounds

The microbial depolymerization strategies discussed in Section 3 release chemically diverse monomers, including lactate, hydroxyalkanoates, succinate, adipate, 1,4-butanediol, and aromatic intermediates. To enable a circular bioeconomy, these monomers need to be redirected through engineered metabolic pathways toward defined, value-added products rather than being fully mineralized or relying solely on native metabolic products (Table 2). Because many of these intermediates converge at central metabolic entry points, we organize the upcycling strategies in this section around three key metabolic nodes: pyruvate, acetyl-CoA/β-oxidation, and aromatic/dicarboxylate nodes. Each node is discussed in terms of the monomers that enter it, engineering strategies that redirect carbon flux, and products that can be reached. This node-based framework highlights how biodegradable plastic–derived carbon can be systematically rewired using synthetic biology and metabolic engineering to generate chemicals, fuels, or next-generation biopolymers.

### 4.1 Valorization through the pyruvate node

Enzymatic depolymerization of PLA releases lactic acid and lactyl oligomers, which are readily assimilated into the central carbon metabolism. Lactate is oxidized to pyruvate via NAD^+^-dependent lactate dehydrogenase (LDH), positioning pyruvate as a metabolic hub for biosynthesis of diverse value-added chemicals. Therefore, redirecting pyruvate-derived carbon toward industrial products, including succinic acid, 1,2-propanediol (1,2-PDO), and other organic acids, has become a central objective of metabolic engineering strategies aimed at PLA upcycling.

A representative example is the engineered conversion of lactate to 1,2-PDO in *Escherichia coli*, in which the introduction of a heterologous pathway enabled *de novo* production of both stereoisomers under controlled fermentation conditions. The engineered strain achieved 17.3 g/L R-1,2-PDO (42.2% molar yield) and 9.3 g/L S-1,2-PDO (23.2% molar yield), demonstrating effective redirection of pyruvate flux toward industrial diol synthesis (Niu et al., 2019). These results illustrate how metabolic rewiring can transform PLA-derived carbon into platform chemicals widely used in polymer and resin production.

Beyond whole-cell fermentation, lactate valorization has also been demonstrated in cell-free systems. LDH-mediated oxidation of lactate generates pyruvate while reducing NAD+ to NADH, functioning simultaneously as both a precursor and an electron donor. Nishikawa and group reported cell-free synthesis of four amino acids, including Asn, Asp, Gln, and Glu, from lactate using the PURE system (PUREfrex) coupled with a one-pot, cofactor-self-sufficient multienzyme cascade supplied with DL-PLA, α-ketoglutarate, CO₂, and NH₄^+^ (Nishikawa et al., 2024). In this cascade, DL-PLA serves a dual role: (i) providing reducing equivalents through LDH-mediated NADH regeneration to drive reductive amination reactions and (ii) supplying pyruvate as a carbon precursor for amino acid biosynthesis (Schulz-Mirbach et al., 2022). Notably, a “PLA-eating” PURE system was subsequently constructed by incorporating mRNA encoding a recently reported PLA hydrolase (IS12; PLAase), enabling *in situ* PLAase synthesis and continuous depolymerization of externally supplied PLA to sustain lactate generation in a feedback loop configuration (Distaso et al., 2023). These advances highlight the potential of integrating depolymerization and biosynthesis within programmable biochemical systems.

Succinic acid represents another major pyruvate-derived platform chemical, relevant to PBS precursor production and solvent markets. Engineered *Mannheimia succiniciproducens* strains, optimized to enhance carbon flux through the reductive branch of the TCA cycle while eliminating competing pathways, have achieved commercially relevant titers in defined media. Targeted modulation of the GltA regulatory node further increased succinate accumulation beyond 100 g/L (Kim et al., 2025). These findings demonstrate the feasibility of coupling lactate assimilation to high-titer C_4_ platform acid production within engineered hosts.

Beyond pyruvate-derived chemicals, lactate released from PLA hydrolysates can be channeled into a family of medium-chain carboxylic acids such as n-butyrate, isobutyrate, n-valerate, and n-caproate, through the acrylate and reverse β-oxidation pathways operative in *Megasphaera elsdenii*, *Megasphaera hexanoica*, *Clostridium kluyveri*, and related organisms (Yoshikawa et al., 2018; Reddy et al., 2018), with successive C2 elongation cycles producing valeryl-CoA and caproyl-CoA before terminal hydrolysis to the corresponding free acids. These short-chain acids are not metabolic curiosities: n-valerate is a precursor for synthetic lubricants and the 3-hydroxyvalerate co-monomer of PHBV, n-butyrate underpins biofuel and polyester intermediate markets, isobutyrate feeds branched ester fragrances and plasticizers, and n-caproate supplies medium-chain ester lubricants and feed additives. The same acids are routinely recovered from food-industry side streams by mixed-culture fermentation, suggesting that lactate from PLA can be processed in the same downstream infrastructure as agro-industrial volatile-fatty-acid streams (den Boer et al., 2016; Kocatürk-Schumacher et al., 2017).

Mixed microbial systems (i.e., consortia) provide an additional strategy for valorizing PLA hydrolysates. Conversion of hydrolyzed PLA into a spectrum of C2–C6 multi-carbon carboxylates can be achieved by using mixed microbial consortia enriched with members of the genera *Caproiciproducens*, *Lactobacillus*, and *Clostridium sensu stricto*. In this system, pretreated PLA food-packaging waste was upcycled into short- and medium-chain carboxylates, with n-butyrate produced as the dominant product at 6.5 g/L (38% yield) (Contreras-Dávila et al., 2019). Such approaches illustrate the potential of community-level metabolism to expand product diversity from lactate-rich streams.

Further improvements in pyruvate-derived product yields have been achieved through dynamic pathway regulation and fermentation optimization. Various strategies enable tighter flux redirection toward target products, including heterologous expression of pyruvate decarboxylases, redox-balancing modules, CRISPR-based transcriptional control, and elimination of competing biomass pathways (Wang T. et al., 2025). Although many of these engineering principles have not yet been broadly implemented using crude PLA hydrolysates as feedstocks, they provide a transferable framework for systematic lactate valorization within integrated biodegradable plastic upcycling platforms.

Collectively, these studies establish that lactic acid released from PLA depolymerization can be efficiently funneled into higher-value chemicals through targeted metabolic rewiring at the pyruvate node. Established strategies, including heterologous pathway insertion, carbon flux redirection, redox balancing, and dynamic regulation, provide a robust foundation for pyruvate-centered bioplastic upcycling. Rather than permitting lactate to dissipate through unmanaged biodegradation, engineered microbial and cell-free systems enable its controlled conversion into industrially relevant products, reinforcing the central role of metabolic bio-funneling in a circular bioeconomy for biodegradable plastics.

### 4.2 Valorization at the acetyl-CoA/β-oxidation node

Biodegradable polyesters that release hydroxy acids, particularly PHA-derived 3-hydroxybutyrate (3HB) and related hydroxyalkanoates, converge metabolically at the acyl-CoA and acetyl-CoA nodes. Following depolymerization, hydroxy acids are activated to hydroxyacyl-CoAs and processed either via thiolase-mediated cleavage or via oxidative pathways that generate acetyl-CoA and TCA/glyoxylate intermediates. This node represents a central control point in circular upcycling because acetyl-CoA supports both (i) re-polymerization into PHAs and (ii) diversion toward higher-value products, including alcohols, ketones, organic acids, and specialty monomers. Importantly, experimental evidence indicates that acetyl-CoA valorization is governed less by simple pathway introduction than by flux competition at TCA entry, intracellular CoA availability, and termination chemistry mediated by thioesterases or CoA transferases, factors that determine whether carbon is retained intracellularly or secreted as product.

Modulation of citrate synthase (GltA), which gates acetyl-CoA entry into the TCA cycle, provides a quantitative illustration of this principle. In engineered *E. coli* expressing *phaA* and *phaB*, chromosomal GltA variants significantly enhanced extracellular (R)-3HB production. The GltA (A267T) variant achieved 4.9 g/L (17% yield), while GltA (K167A) reached 4.6 g/L (16% yield), demonstrating that central metabolic tuning, rather than additional enzyme installation alone, controls carbon partitioning into PHA-relevant hydroxyacids (Rajpurohit and Eiteman, 2024). These results reinforce acetyl-CoA gating as a decisive regulatory lever for hydroxy acid secretion.

Extension of acetyl-CoA through reverse β-oxidation further expands product scope toward medium-chain compounds (C6–C10) aligned with biodegradable polyester chemistry. Integration of reverse β-oxidation with *ω*-oxidation modules enabled production of >0.8 g/L (8.8% yield) total C6–C10 ω-hydroxy acids, which were subsequently oxidized to ~0.5 g/L dicarboxylic acids in minimal medium. Notably, 6-hydroxyhexanoic acid and adipic acid, key intermediates in biodegradable polymer synthesis, were among the products, demonstrating the utility of acetyl-CoA extension platforms even when plastic-derived substrates are not directly supplied (Clomburg et al., 2015). This strategy highlights how central carbon elongation modules can interface with the production of polyester-relevant monomers.

A direct example of coupling depolymerization with acetyl-CoA-linked valorization is observed in polycaprolactone (PCL) processing. Enzymatic depolymerization of PCL using *Candida antarctica* lipase B (CALB) releases 6-hydroxyhexanoic acid (6-HHA), which can be oxidized to adipic acid (AA) in engineered *E. coli* expressing heterologous 6-HHA dehydrogenase (*chnD*) and 6-oxohexanoic acid dehydrogenase (*chnE*) from *Acinetobacter* strain SE19 (Oh Y.-R. et al., 2022). After 16 h of batch fermentation, AA reached 2.92 g/L with 100% yield, yielding 0.18 g/L/h. Subsequent process optimization revealed toxicity limitations associated with 6-HHA, constraining batch titers to ~4 g/L. However, controlled fed-batch feeding at 0.22 g/L/h increased AA production to 15.6 g/L, representing a 4.2-fold improvement over batch conditions. Downstream, adipic acid can re-enter central metabolism through β-oxidation, generating acetyl-CoA and succinyl-CoA that feed directly into the TCA cycle, thereby reinforcing metabolic circularity.

Enzymatic depolymerization of polyethylene furanoate (PEF), a next-generation bio-based polyester developed as a sustainable alternative to PET, releases the monomeric intermediates EG and FDCA (Kumar et al., 2024). An engineered EG valorization pathway has been established in *E. coli* by heterologously expressing the alcohol dehydrogenase Gox0313 and overexpressing the endogenous aldehyde dehydrogenase AldA, while eliminating competing reductive pathways via CRISPR-Cas9 deletion of fucO and yqhD. This strategy redirected EG oxidation toward glycolic acid (GA) production, achieving a 3.78 g L^−1^ titer from 5.62 g L^−1^ EG, with a yield of 0.67 g g^−1^ (Gong et al., 2025). Yeast systems have also demonstrated strong potential for EG conversion. A recent study reported a two-step bioconversion of EG to glycolic acid in *Saccharomyces cerevisiae*, in which process optimization using a design-of-experiments approach enabled the production of 4.51 ± 0.12 g L^−1^ GA with a conversion efficiency of 94.25 ± 1.74% from 6.21 ± 0.04 g L^−1^ EG. Among the strains evaluated in that study, the non-conventional yeast *Scheffersomyces stipitis* exhibited the highest glycolic acid production, reaching 23.79 ± 1.19 g L^−1^ with a yield of 76.68% (Senatore et al., 2024).

Acetyl-CoA engineering is equally critical for re-polymerization platforms. In *Saccharomyces cerevisiae* expressing a bacterial PHB biosynthesis pathway, enhancement of cytosolic acetyl-CoA availability during ethanol metabolism increased PHB titers to 43.11 mg/L (13% yield), compared to 1.85 mg/L in control strains (Kocharin et al., 2012). Although titers remain below industrial thresholds, this work establishes a key design rule: hydroxy acid-derived carbon must be coupled to compartment-specific acetyl-CoA supply and balanced redox metabolism to enable effective intracellular polymer synthesis.

Among PHA copolymers, PHBV deserves specific mention because its lower crystallinity and broader processing window make it a more practical drop-in alternative to polypropylene than PHB alone (Policastro et al., 2021). PHBV is biosynthesized by *Cupriavidus necator* and related organisms via the PhaA/PhaB/PhaC pathway. Acetyl-CoA supplies the 3-hydroxybutyrate (3HB) units, while propionyl-CoA generated from valerate, propionate, or odd-chain co-feeds supplies the 3-hydroxyvalerate (3 HV) co-monomer; the 3 HV mole fraction is tunable up to ~30 mol% by adjusting the propionyl-CoA/acetyl-CoA ratio (Cai F. et al., 2023). PHBV depolymerization releases 3-hydroxybutyrate (entering the acetyl-CoA node) and 3-hydroxyvalerate (entering propionyl-CoA metabolism), linking PHA re-polymerization to the n-valerate chemistry described above in Section 4.1 and supporting closed-loop resynthesis of PHBV with controlled 3 HV content.

Collectively, these studies position acetyl-CoA as a controllable metabolic gate that determines carbon allocation between secretion, chain extension, and re-polymerization. Rather than serving as a passive intermediate, the acetyl-CoA/β-oxidation node functions as a strategic engineering leverage point for balancing monomer recovery and higher-value product synthesis, making it central to circular polyester upcycling strategies.

### 4.3 Aromatic and dicarboxylate node

Depolymerization of PBAT generates a chemically mixed monomer stream composed primarily of adipic acid (AA) and 1,4-butanediol (BDO), with terephthalic acid (TPA) representing the aromatic fraction. Similarly, depolymerization of related polyesters such as PBT and PBS releases BDO as a recurring aliphatic unit. From an upcycling perspective, AA and BDO function as high-flux aliphatic entry points that feed central metabolism through β-oxidation and gluconeogenic routes, whereas TPA serves as a benchmark aromatic dicarboxylate requiring dedicated catabolic modules (Op de Hipt et al., 2025). This node, therefore, represents a metabolic interface where aliphatic hydrolysates can be redirected toward aromatic building blocks by supplying precursors to the shikimate pathway.

Engineering efforts in *Pseudomonas taiwanensis* illustrate this concept. The tyrosine-overproducing platform strain *P. taiwanensis* GRC3Δ5-TYR2 was adapted for BDO-based aromatic production. In evolved strains, a mutant ethanol dehydrogenase encoded by PVLB_10,545 (functionally substituting PedE from *Pseudomonas putida* KT2440) catalyzed the initial oxidation of BDO, while a mutation in PVLB_12690 accelerated a rate-limiting downstream step following 4-hydroxybutyrate. These modifications enabled growth on BDO as the sole carbon source. However, a clear trade-off between growth and production was observed: mutations that enhanced AA assimilation reduced aromatic overproduction, highlighting flux-partitioning constraints at the dicarboxylate–aromatic interface (Op de Hipt et al., 2025). Integration of the tyrosine ammonia-lyase (RpcTAL) module at the Tn7 site enabled *de novo* production of 4-coumarate with a 14.4 ± 0.1% (Cmol/Cmol) yield. In this pathway, 4-hydroxybutyrate is oxidized by PVLB_12675 and possibly PVLB_13315 to succinate semialdehyde, which is further converted to succinate via the succinate semialdehyde dehydrogenase Sad-II, linking BDO oxidation to central metabolism.

Adipic acid assimilation provides a complementary entry route. Heterologous expression of the *dcaAKIJP* operon in *Pseudomonas putida* KT2440, coupled with deletion of PaaXY, first enabled growth on AA as the sole carbon source. Adipic acid is activated to adipyl-CoA and subsequently metabolized via β-oxidation. Adaptive laboratory evolution (ALE) and reverse engineering of the AA-metabolizing base strain (KT2440ge) generated the final strain KT2440ge *ΔPpaaF*-*paaYX*: P14g Δ*psrA*, which accumulated 25.3 ± 4.2% PHA with a carbon yield of 9.2% (g/g carbon) from 27.1 mM adipic acid (Op de Hipt et al., 2025). Extending this strategy, an evolved and engineered strain of *P. taiwanensis* harboring *dcaAKIJP* in *paaYX* and *ΔpsrA4* produced 4-coumarate from adipic acid with a carbon yield of 11.5 ± 0.3% (C mol/C mol), further demonstrating that dicarboxylate assimilation can be coupled to aromatic biosynthesis (Op de Hipt et al., 2025).

Terephthalic acid metabolism represents the dedicated aromatic arm of this node. Identification of a TPA transporter, together with transcriptomic elucidation of TPA depolymerization pathways in *Rhodococcus* sp., enabled assembly of modular TPA catabolic systems. TPA is converted to protocatechuate (PCA), a valuable aromatic intermediate, which can be further hydroxylated at the meta position to gallic acid (GA) using a mutant p-hydroxybenzoate hydroxylase from *P. putida* KT2440 (Hosaka et al., 2013). Implementation of a two-strain *E. coli* consortium, one strain converting TPA to PCA and the second converting PCA to GA, achieved a 92.5% molar yield. Beyond GA production, additional downstream pathways enable the synthesis of other high-value aromatic chemicals. One example is pyrogallol, which can be produced from TPA through two potential routes: decarboxylation of GA or hydroxylation of catechol derived from PCA decarboxylation. The catechol hydroxylation route is generally favored because gallic acid decarboxylase (LpdC) exhibits promiscuous activity that leads to catechol accumulation rather than efficient pyrogallol formation. To overcome this limitation, a synthetic pyrogallol biosynthesis pathway was constructed in *E. coli* by integrating a PCA decarboxylation module that converts PCA to catechol with a catechol hydroxylation module encoded by *PhKLMNOPQ*, enabling conversion of catechol to pyrogallol in either single-strain or distributed two-strain systems (Wang J. et al., 2018). In addition to hydroxylation-based routes, catechol generated from TPA through the PCA intermediate can also undergo ring-cleavage reactions to produce *cis,cis*-muconic acid (MA), a versatile dicarboxylic acid precursor for adipic acid and other polymer intermediates (Johnson et al., 2016; Han et al., 2015).

For PBAT/PBS hydrolysates, a strategic objective is expansion of the “dicarboxylate/diol → central metabolism → aromatic product” framework while maintaining tolerance to mixed monomer streams. Proof-of-concept implementation integrated a tyrosine ammonia-lyase module to produce 4-coumarate from either AA or BDO. Under optimized induction conditions, engineered strains achieved ~3 mM 4-coumarate, corresponding to 14.4% ± 0.1% (C mol/C mol) from BDO and 11.5% ± 0.3% from AA. A mixed-culture configuration enabled simultaneous utilization of both substrates with an overall yield of 14.0% ± 0.1% (Op de Hipt et al., 2025). These results highlight the importance of balancing substrate activation, redox flux, and gluconeogenic precursor supply to overcome competition between biomass formation and aromatic overproduction.

Collectively, these studies establish the aromatic and dicarboxylate node as a critical metabolic interface for polyester upcycling. Efficient PBAT/PBS valorization requires coordinated control of substrate transport, CoA activation, oxidative entry into central metabolism, gluconeogenic flux redistribution, and tolerance to chemically heterogeneous hydrolysates. When these elements are aligned, aliphatic monomers derived from biodegradable plastics can be converted into higher-value aromatic building blocks rather than dissipated through catabolism.

Beyond catabolism and anabolism, overflow metabolism represents a third, growth-rate-dependent mode of carbon use that critically constrains plastic-monomer upcycling. At elevated specific growth rates, cells excrete acetate, lactate, or other partially oxidized intermediates even under fully aerobic conditions. This phenotype arises jointly from growth optimization and population-level proteome heterogeneity (Wang, 2025). When polymer hydrolysates supply monomers near the host’s overflow threshold, carbon-conversion efficiency drops at exactly the operating point that maximizes volumetric productivity. Mitigation strategies include feed-rate control, attenuation of overflow-pathway genes (e.g., pta-ackA in *E. coli*), and consortium-based division of labor.

### 4.4 Microbial consortia

Monoculture engineering often encounters intrinsic limitations when processing heterogeneous bioplastic hydrolysates. PBAT and PBS release chemically distinct monomers (adipate, BDO, and terephthalate), while PLA and PHA streams generate hydroxy acids with differing redox states (Qiu et al., 2024). Consolidating depolymerization, assimilation, and product formation within a single chassis frequently imposes metabolic burden, redox imbalance, and pathway interference. Microbial consortia address this constraint through division of labor, distributing substrate conversion and biosynthesis across specialized strains.

A foundational blueprint for enzyme-microbe coupling was demonstrated by Tournier and group, in which enzymatically liberated PET monomers were biologically valorized, enabling repolymerized PET and aromatic intermediates under controlled conditions. Although PET is not biodegradable, this two-stage architecture depolymerization, followed by engineered microbial conversion, validated the feasibility of integrating polymer breakdown with biosynthesis (Tournier et al., 2020).

More directly relevant to biodegradable streams, engineered *Pseudomonas taiwanensis* co-cultures optimized separately for adipate and BDO assimilation achieved simultaneous substrate utilization and ~14% C mol/C mol yield to 4-coumarate under mixed-monomer conditions (Op de Hipt et al., 2025). Another experimentally validated consortium uses an engineered *E. coli*-*P. putida* system to distribute metabolic functions and improve mixed-feedstock conversion, achieving 1.30 g/L mcl-PHA (3% g/g) from mixed sugars and 1.02 g/L (2% g/g) mcl-PHA from lignocellulosic hydrolysate (Qin et al., 2022).

Beyond titers, consortia confer three structural advantages: reduced heterologous burden per strain, improved redox balancing through metabolic compartmentalization, and enhanced tolerance to toxic intermediates via specialized detoxification modules. Synthetic ecology studies further demonstrate that engineered two-member systems can maintain stable population ratios over extended cultivation when nutrient partitioning is rationally designed, reinforcing the feasibility of sustained operation (Ziesack et al., 2019; Kerner et al., 2012).

A balanced view of upcycling also requires comparing engineered systems against open or defined mixed microbial cultures, which have been deployed industrially for decades in anaerobic digestion, activated sludge treatment, and volatile-fatty-acid production from food and forestry side streams (Hakalehto et al., 2025; den Boer et al., 2016). Mixed cultures offer intrinsic tolerance to feedstock heterogeneity, compatibility with non-aseptic operation that substantially lowers capital and operating costs by eliminating sterilization infrastructure and bioreactor jacket-cooling demand (Khedkar et al., 2025), and ready integration with existing biorefineries and wastewater treatment infrastructure (Hakalehto, 2025). Their main limitations are reduced product specificity and the difficulty of routing carbon into a single high-value monomer rather than a mixture of short-chain acids.

Engineered monocultures and rationally designed consortia, by contrast, deliver predictable yields and access to non-natural targets such as *cis,cis*-muconate from PET- or lignin-derived aromatics (Sonoki et al., 2018; Kohlstedt et al., 2022), 2,3-butanediol (Lee et al., 2021), and engineered repolymerization precursors, at the cost of sterilization requirements, GMO regulatory burden, and the residual concern that engineered networks may behave unexpectedly when interacting with native microbial, plant, and animal communities; biosafety implications of these scenarios are discussed in Section 6.6. In practice, the two strategies are likely to converge through hybrid pipelines in which a defined engineered consortium is operated downstream of a mixed-culture pre-fermentation step, combining mixed-culture robustness with engineered specificity.

**Figure 4:**
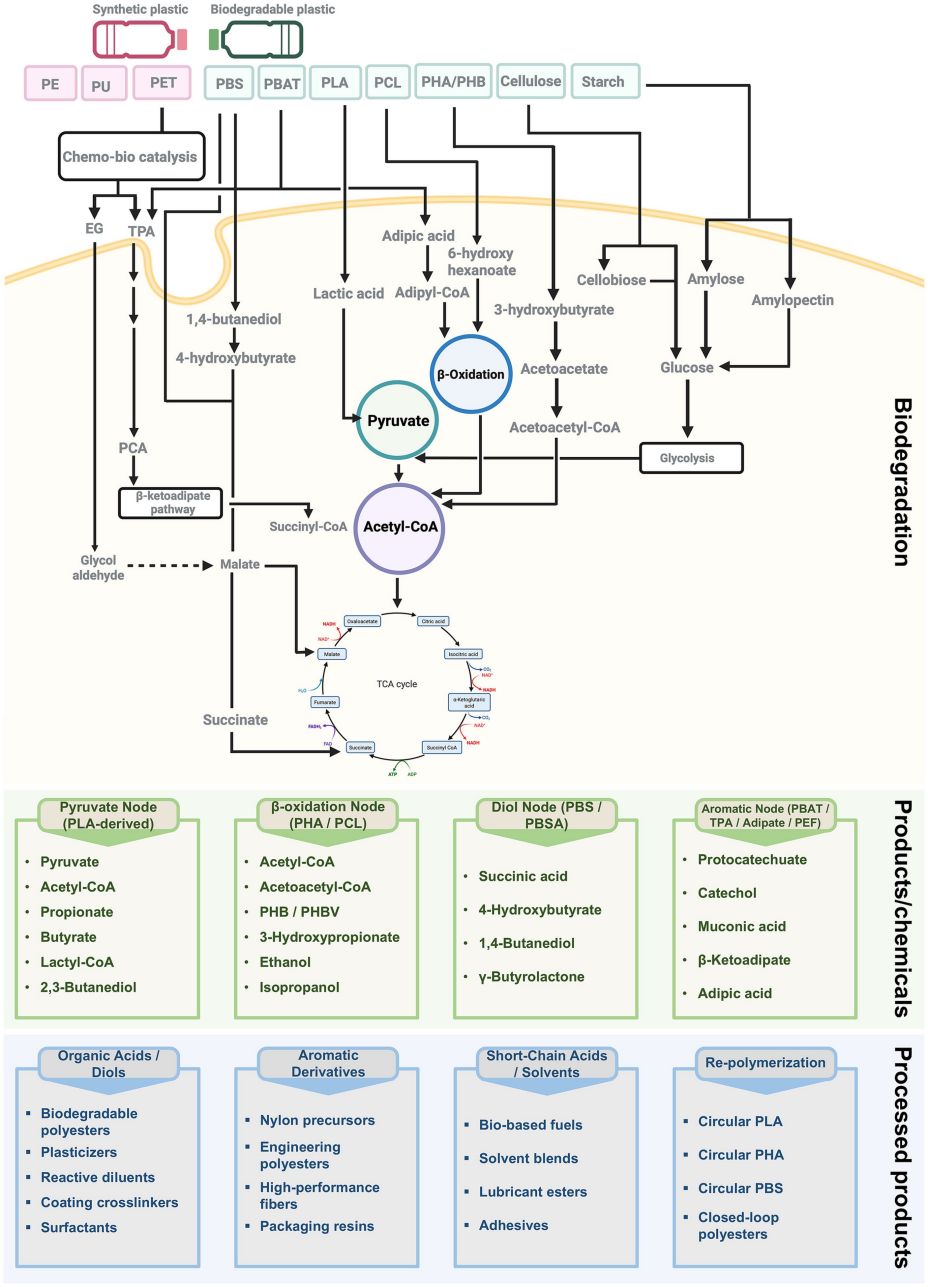
Engineered microbial upcycling framework for biodegradable and selected synthetic plastics. Schematic overview of enzymatic biodegradation, metabolic bio-funneling, and downstream valorization of biodegradable polymers. Polymer-derived monomers enter central metabolic nodes (pyruvate, β-oxidation/acetyl-CoA, diol, and aromatic pathways), where engineered microbial systems convert them into platform chemicals and industrial intermediates. These are further processed into market-relevant materials, including biodegradable polyesters, specialty chemicals, fuels, and closed-loop circular polymers. The framework illustrates how controlled bioconversion can redirect biodegradable plastic waste from passive environmental degradation toward integrated circular manufacturing systems. Detailed enzymatic mechanisms and metabolic pathway diagrams corresponding to each node are provided in Figure 5.

**Figure 5:**
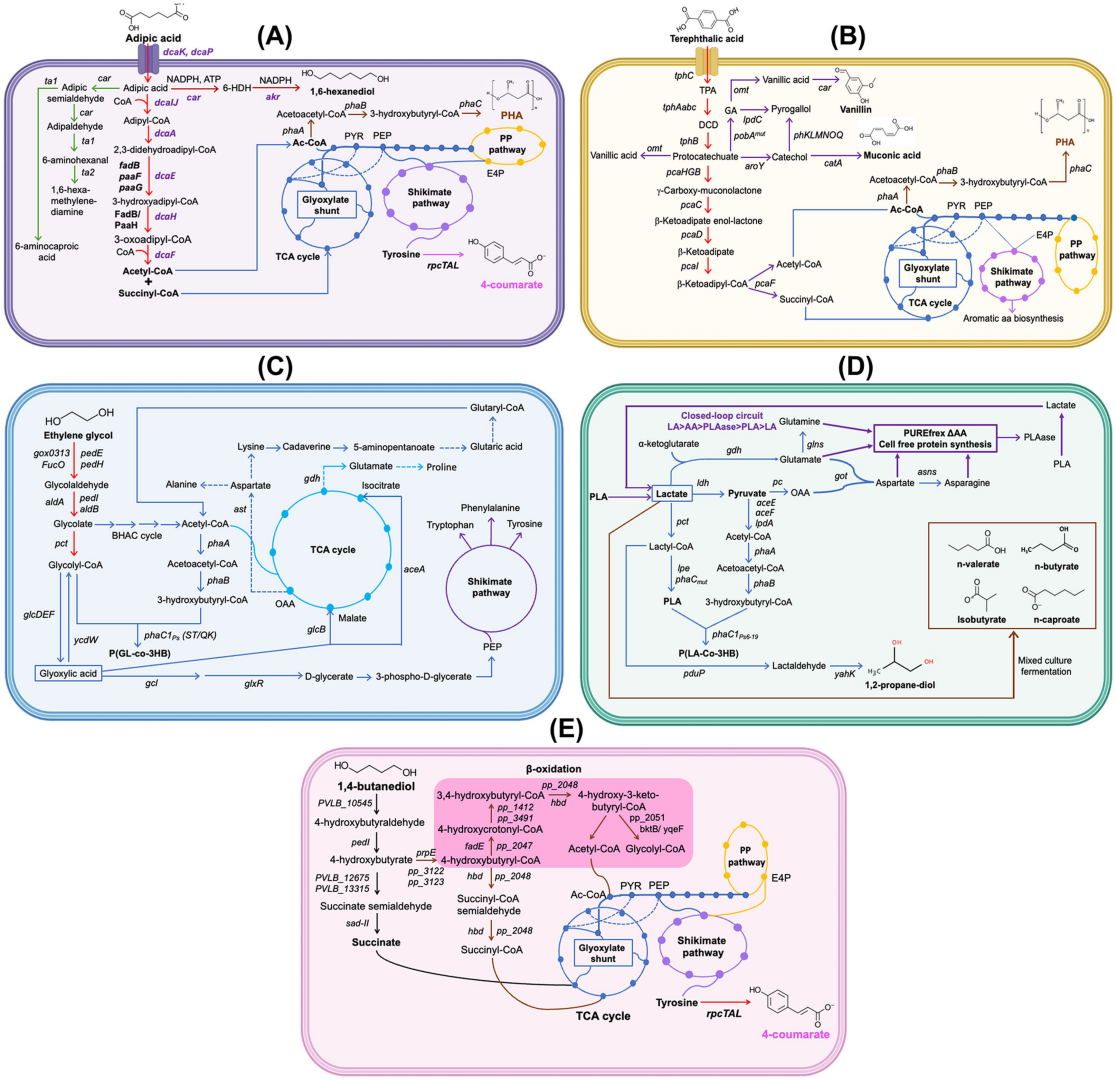
Detailed metabolic pathway maps for microbial upcycling of biodegradable and selected synthetic plastic-derived monomers. The figure expands the conceptual framework presented in Figure 4 by detailing representative enzymatic and metabolic steps in which polymer-derived monomers are assimilated and converted into value-added products by engineered microorganisms. **(A)** Bio-upcycling of adipic acid: The metabolic map showing the genes involved in Adipic acid (AA) catabolism and synthesis of upcycled products. AA catabolism genes are clustered in two operons. In the operon *dcaAKIJP*, *dcaK* and *dcaP* code for the transporter DcaK and DcaP, which uptake AA in microbial cells. In the next step, CoA transferase (*dcaIJ*) converts adipic acid to adipyl-CoA, which is further converted to 2,3-didehydroadipyl-CoA by the acyl-CoA dehydrogenase (*dcaA*). The other operon *(dcaECHF)* encodes enzymes related to β-oxidation, including an enoyl-CoA hydratase (*dcaE*), ketoacyl-CoA reductase (*dcaC*), hydroxyl-CoA dehydrogenase (*dcaH*), and a thiolase (*dcaF*), which converts 2,3-didehydroadipyl-CoA to acetyl-CoA and succinyl-CoA to enter the TCA cycle. Acetyl-CoA is converted to PHA by a 3-step pathway consisting of acetyl-CoA acetyltransferase (*phaA*), acetoacetyl-CoA reductase (*phaB*), and PHA synthase (*phaC*). Acetyl-CoA, through the TCA cycle, the pentose phosphate pathway, and the shikimate pathway, produces tyrosine, which gets converted to Coumarate by the Tyrosine Ammonia Lyase from *Rivularia* sp. PCC 7116 (*rpcTAL*). **(B)** Bio-upcycling of terephthalic acid (TPA); TPA catabolism pathway genes are encoded by two operons. In the first operon, TphC (*tphC*) functions as a transporter to uptake TPA into the cells. In the next step, TPA is converted to 1,2-dihydroxy-3,5-cyclohexadiene-1,4-dicarboxylate (DCD) by TPA 1,2-dioxygenase (*tphAabc*), and then to protocatechuate by DCD dehydrogenase (t*phB*). Protocatechuate is either converted to multiple aromatic products or enters the β-oxidation pathway to produce acetyl-CoA and succinyl-CoA. *pobA*: p-hydroxybenzoate hydroxylase; *lpdC*: gallic acid decarboxylase; *omt*: O-methyltransferase; *car*: carboxylic acid reductase; *aroY*: PCA decarboxylase; *catA*: Catechol 1,2-dioxygenase; *phKLMNOQ:* Phenol hydroxylase; *pcaHGB*: protocatechuate 3,4-dioxygenase and 3-carboxy-cis,cis-muconate cycloisomerase; *pcaC*: *γ*-Carboxy-muconolactone decarboxylase; *pcaD*: β-Ketoadipate enol-lactone hydrolase; *pcaI*: β-Ketoadipate succinyl-CoA transferase; *pcaF*: *β*-ketoadipyl-CoA thiolase; Acetyl-CoA and succinyl-CoA enters into the central carbon metabolism through TCA cycle. Acetyl-CoA, through the PHA biosynthesis route, is converted to PHA by a 3-step pathway consisting of acetyl-CoA acetyltransferase (*phaA*), acetoacetyl-CoA reductase (*phaB*), and PHA synthase (*phaC*). **(C)** Bio-upcycling of PEF monomer ethylene glycol (EG); EG is first converted to glycolaldehyde by alcohol dehydrogenase (*fucO* or gox0313 in *E.coli; pedE/pedH in Pseudomonas putida KT2440*), which then gets converted to glycolate by aldehyde dehydrogenase (*aldA* in *E.coli*; *pedI*/*aldB* in *P. putida* KT2440). Glycolate, by the activity of propionyl-CoA transferase (*pct*), forms Glycolyl-CoA and is further converted to Glyoxylic acid by glycolate oxidase (*glcDEF*). Glyoxylic acid gets converted to malate-by-malate synthase (*glcB*) or to isocitrate by isocitrate lyase (*aceA*) to enter the TCA cycle. Glycolate through the BHAC cycle gets converted to Acetyl-CoA, which is converted to 3-hydroxybutyryl-CoA (3-HB) in 2 steps by acetyl-CoA acetyl transferase (*phaA*) and acetoacetyl-CoA reductase (*phaB*). The mutant PHA synthase (*phaC1_Ps_ (ST/QK)*) co-polymerizes glycolyl-CoA and 3-HB to form P(GL-co-3HB). The intermediate metabolites in the shikimate pathway produce aromatic amino acids. TCA intermediate oxaloacetate (OAA) and *α*-ketoglutarate enter the amino acid biosynthesis. *Gdh*: glutamate dehydrogenase; *ast*: aspartate aminotransferase. **(D)** Bio-upcycling of PLA; PLA monomer lactate is converted to lactyl-CoA by propionyl-CoA transferase (*pct*), which is converted back to PLA by lactate polymerizing enzyme (*lpe*) mutant variant of *phaC*. Lactate, by a single-step reaction by lactate dehydrogenase (*ldh*), is converted to pyruvate, which is converted to acetyl-CoA by the pyruvate dehydrogenase complex (*aceE, aceF, lpdA*). Acetyl-CoA is converted to 3-hydroxybutyryl-CoA (3-HB) in 2-steps by acetyl-CoA acetyltransferase (*phaA*) and acetoacetyl-CoA reductase (*phaB*). In the next step, PLA and 3-HB are copolymerized into P(LA-Co-3HB) by a mutant version of PHA synthase (*phaC1_Ps6–19_*). In a separate pathway, lactate is converted to lactaldehyde by CoA-dependent lactaldehyde dehydrogenase (*pduP*), which is further converted to 1,2-propanediol by lactaldehyde reductase (*yahK*). Lactate through the pyruvate node can enter the amino acid biosynthesis and further synthesize PLAase enzyme by the cell-free protein synthesis system PUREfrex to create a Closed-loop circuit (LA > AA>PLAase>PLA > LA) for PLA depolymerization. In parallel, lactate released from PLA depolymerization can be channeled into n-butyrate, isobutyrate, n-valerate, and n-caproate through the acrylate pathway and reverse β-oxidation/chain-elongation cycles operative in mixed cultures containing *Megasphaera elsdenii*, *M. hexanoica*, and *Clostridium kluyveri*, providing access to short-chain platform acids alongside the canonical engineered products shown above. *pc*: Pyruvate carboxylase; *got*: glutamic-oxaloacetic transaminase; *gdh*: glutamate dehydrogenase; *glns*: Glutamine synthetase; *asns*: Asparagine synthase. **(E)** Bio-upcycling of 1,4-butanediol (BDO); BDO enters the pathway through the conversion into 4-hydroxybutyraldehyde by alcohol dehydrogenase (*PVLB 10545*), which gets converted to 4-hydroxybutyrate by aldehyde dehydrogenase (*pedI*). This 4-hydroxybutyrate, through β-oxidation, is converted into acetyl-CoA and glycolyl-CoA. *PVLB_13315*, annotated as a 3-hydroxybutyrate dehydrogenase, and PVLB_12675 convert 4-hydroxybutyrate to Succinate semialdehyde, which then forms succinate by the activity of succinate semialdehyde dehydrogenase (*sad-II*). 4-hydroxybutyrate by the activity of CoA-ligase/transferase (*prpE, pp_3122, pp_3123*) gets converted into 4-hydroxybutyryl-CoA, which, by a 4-step reaction, forms acetyl-CoA to enter the central carbon metabolism. *pp_2047*/ *fadE: acyl-CoA dehydrogenase; pp_1412/ pp_3491:* Enoyl-CoA hydratase; pp_2048/ hbd: 3-hydroxyacyl-CoA dehydrogenase; *pp_2051*/*bktB*/*yqeF*: acetyl-CoA C-acetyltransferase. Succinyl-CoA semialdehyde, formed through the β-oxidation, is converted to succinyl-CoA by the 3-hydroxyacyl-CoA dehydrogenase. Succinate and succinyl-CoA enter the TCA cycle. Acetyl-CoA, formed through the TCA cycle, the pentose phosphate pathway, and the shikimate pathway, produces tyrosine, which gets converted to Coumarate by the Tyrosine Ammonia Lyase from *Rivularia* sp. PCC 7116 (*rpcTAL*). Another pathway converts AA to 1,6-hexanediol by the activity of carboxylate reductases (*car*) and aldehyde-keto reductase (*akr*). AA gets converted to 6-aminocaproic acid in a 2-step reaction by carboxylate reductase (*car*) and *ω*-transaminase 1 (*ta1*). Following another route, the common intermediate adipic semialdehyde is converted to 1,6-hexamethylene diamine. *ta2* = ω-transaminase 2.

A consolidated overview of experimentally validated upcycling systems, organized by metabolic entry node, including reported titers and yields, is provided in Table 3. An overview of enzymatic biodegradation, metabolic bio-funneling, and downstream valorization of biodegradable polymers is presented in Figure 4, while Figure 5 provides detailed metabolic pathway maps illustrating representative enzymatic and microbial routes connecting plastic depolymerization products to value-added chemical synthesis.

**Table 3:** Engineering approaches to bioplastic upcycling: a node-based summary of microbial and cell-free systems for redirecting biodegradable-plastic-derived monomers into value-added products.

Metabolic node	Host/biocatalyst	Substrate/entry point	Engineering strategy	Product(s)	Reported performance	Representative applications	Indicative market size	References
Pyruvate node	*Escherichia coli* (heterologous 1,2-PDO pathway)	PLA-derived lactate → pyruvate	Heterologous 1,2-propanediol pathway insertion; stereoselective fermentation control	(R)-1,2-propanediol; (S)-1,2-propanediol	(R): 17.3 g L^−1^, 42.2% molar yield; (S): 9.3 g L^−1^, 23.2% molar yield	Solvents, polymer and resin production, optically active polyesters, pharmaceutical and agrochemical intermediates	5.4–5.9 B	Niu et al. (2019)
Pyruvate node	Cell-free PURE system (PUREfrex) with multienzyme cascade	DL-PLA-derived lactate; α-ketoglutarate, CO₂, NH₄^+^	Cofactor-self-sufficient one-pot enzyme cascade; LDH-mediated NADH regeneration; in situ PLAase (IS12) expression for feedback depolymerization	Amino acids (Asn, Asp., Gln, Glu)	Four amino acids are synthesized; lactate serves as both a carbon precursor and an electron donor	Programmable biosynthesis; integrated depolymerization-biosynthesis systems; specialty biochemicals	n.r.	Nishikawa et al. (2024), Schulz-Mirbach et al. (2022) and Distaso et al. (2023)
Pyruvate node	*Mannheimia succiniciproducens*	PLA-derived lactate → pyruvate → oxaloacetate	Reductive TCA-branch flux enhancement; competing-pathway deletion; GltA regulatory-node modulation	Succinic acid	>100 g L^−1^ in defined medium	PBS precursor, biopolymer production, solvents, food-grade acidulants, resins and coatings, chemical intermediates (1,4-BDO, THF)	190–200 M	Kim et al. (2025)
Pyruvate node	*Megasphaera elsdenii*, *Megasphaera hexanoica*, *Clostridium kluyveri*	PLA-derived lactate	Acrylate and reverse β-oxidation pathways; successive C2 chain-elongation cycles	n-Butyrate, isobutyrate, n-valerate, n-caproate	Chain-elongated short- and medium-chain carboxylic acids (see mixed-culture row for titres)	Synthetic lubricants, PHBV 3 HV co-monomer, biofuels, polyester intermediates, branched ester fragrances, plasticizers, feed additives	0.17–0.20 B (n-butyrate)	Yoshikawa et al. (2018) and Reddy et al. (2018)
Pyruvate node	Mixed microbial consortium (*Caproiciproducens*, *Lactobacillus*, *Clostridium sensu stricto*)	Hydrolyzed PLA food-packaging waste	Community-level chain elongation; mixed-culture (non-aseptic) fermentation	C2–C6 carboxylates; n-butyrate dominant	n-Butyrate 6.5 g L^−1^, 38% yield	Animal-feed additives, food flavoring and preservatives, specialty pharmaceutical and cosmetic ingredients, esters, coatings, and fragrances	0.17–0.20 B	Contreras-Dávila et al. (2019)
Pyruvate node	*Escherichia coli* (generalizable dynamic-regulation framework)	Lactate/pyruvate	Heterologous pyruvate decarboxylases; redox-balancing modules; CRISPR-based transcriptional control; competing-pathway elimination	Diverse pyruvate-derived products	n.r. (transferable framework; not yet broadly validated on crude PLA hydrolysates)	Platform chemicals; integrated bioplastic-upcycling pipelines	n.r.	Wang T. et al. (2025)
Acetyl-CoA / β-oxidation node	*Escherichia coli* (phaA, phaB; chromosomal GltA variants)	Acetyl-CoA (PHA-derived 3HB chemistry)	Citrate synthase (GltA) variant tuning to gate acetyl-CoA entry into the TCA cycle	(R)-3-hydroxybutyrate	GltA(A267T): 4.9 g L^−1^, 17% yield; GltA(K167A): 4.6 g L^−1^, 16% yield	Biodegradable PHB/PHA polymers for packaging and medical use, chiral synthons, and tailored hydroxyalkanoate materials	0.25–1.2 B	Rajpurohit and Eiteman (2024)
Acetyl-CoA/β-oxidation node	*Escherichia coli* (reverse β-oxidation with ω-oxidation modules)	Acetyl-CoA elongation	Integration of reverse β-oxidation with ω-oxidation for medium-chain carbon elongation	C6–C10 ω-hydroxyacids; C6–C10 dicarboxylic acids	>0.8 g L^−1^ total ω-hydroxyacids, 8.8% yield; ~0.5 g L^−1^ total diacids	Biodegradable polyester precursors, nylon, bio-based plasticizers, lubricant esters, crosslinking resins, adhesives, controlled-release polymers	7–8 B (dicarboxylic acids)	Clomburg et al. (2015)
Acetyl-CoA/β-oxidation node	*Candida antarctica* lipase B (CALB); engineered *Escherichia coli*; *Acinetobacter* sp. SE19 enzymes	PCL-derived 6-hydroxyhexanoic acid	Enzymatic PCL depolymerization coupled to heterologous 6-HHA dehydrogenase (chnD) and 6-oxohexanoate dehydrogenase (chnE); fed-batch toxicity management	Adipic acid	Batch: 2.92 g L^−1^, 100% yield, 0.18 g L^−1^ h^−1^; fed-batch: 15.6 g L^−1^ (4.2-fold improvement)	Biodegradable polyester monomers, surfactants and emulsifiers, plasticizers, lubricant esters, coating crosslinkers, fragrance intermediates, drug-delivery carriers	6.4–9.4 B	Oh J. Y. et al. (2022)
Acetyl-CoA / β-oxidation node	*Escherichia coli* (Gox0313 alcohol dehydrogenase; AldA aldehyde dehydrogenase)	PEF-derived ethylene glycol	Heterologous Gox0313 expression and AldA overexpression; CRISPR-Cas9 deletion of competing fucO and yqhD	Glycolic acid	3.78 g L^−1^ from 5.62 g L^−1^ EG; 0.67 g g^−1^ yield	Biodegradable polyester (PGA) precursor, cosmetics, specialty chemical intermediate	0.4–0.5 B	Gong et al. (2025) and Kumar et al. (2024)
Acetyl-CoA / β-oxidation node	*Saccharomyces cerevisiae*; *Scheffersomyces stipitis*	PEF-derived ethylene glycol	Two-step EG-to-glycolic-acid bioconversion; design-of-experiments process optimization; non-conventional yeast screening	Glycolic acid	*S. cerevisiae*: 4.51 g L^−1^, 94.25% conversion; *S. stipitis*: 23.79 g L^−1^, 76.68% yield	Biodegradable polyester precursor, cosmetics, specialty chemicals	0.4–0.5 B	Senatore et al. (2024)
Acetyl-CoA / β-oxidation node	*Saccharomyces cerevisiae* (heterologous bacterial PHB pathway)	Acetyl-CoA (cytosolic, from ethanol metabolism)	Enhancement of cytosolic acetyl-CoA availability with redox balancing for re-polymerization	Polyhydroxybutyrate (PHB)	43.11 mg L^−1^, 13% yield (vs 1.85 mg L^−1^ control)	Biodegradable packaging, biomedical applications, closed-loop PHA re-polymerization	50–60 M	Kocharin et al. (2012)
Acetyl-CoA / β-oxidation node	*Cupriavidus necator* and related organisms	Acetyl-CoA (3HB) and propionyl-CoA (3 HV) from valerate/propionate/odd-chain co-feeds	PhaA/PhaB/PhaC pathway; tuning of the propionyl-CoA/acetyl-CoA ratio to control 3 HV incorporation	Poly(3-hydroxybutyrate-co-3-hydroxyvalerate) (PHBV)	3 HV mole fraction tunable up to ~30%	Drop-in biodegradable alternative to polypropylene; packaging; closed-loop PHBV resynthesis	0.25–1.2 B (PHA)	Policastro et al. (2021) and Cai F. et al. (2023)
Aromatic / dicarboxylate node	*Pseudomonas taiwanensis* GRC3Δ5-TYR2 (evolved BDO-utilizing strain)	PBAT/PBS-derived 1,4-butanediol	Mutant ethanol dehydrogenase (PVLB_10,545); PVLB_12690 mutation relieving a downstream bottleneck; RpcTAL tyrosine ammonia-lyase module at Tn7	4-Coumarate	14.4% (C mol/C mol) yield from BDO	Antioxidants and preservatives, anti-inflammatory and antimicrobial agents, flavonoid and stilbene precursors, vanillin precursor, cosmetics, biopesticides	8.8–9.8 M	Op de Hipt et al. (2025)
Aromatic / dicarboxylate node	*Pseudomonas putida* KT2440 (ALE-evolved strain KT2440ge ΔPpaaF-paaYX: P14g ΔpsrA)	PBAT-derived adipic acid	Heterologous dcaAKIJP operon; PaaXY deletion; adaptive laboratory evolution and reverse engineering	Medium-chain-length PHA	25.3% PHA accumulation; 9.2% (g/g) carbon yield from 27.1 mM adipic acid	Flexible biodegradable polymers, elastomeric materials, polymer blends	0.10–0.12 B	Op de Hipt et al. (2025)
Aromatic / dicarboxylate node	Engineered *Pseudomonas taiwanensis* (dcaAKIJP in paaYX, ΔpsrA4)	PBAT-derived adipic acid	Coupling of dicarboxylate assimilation (β-oxidation) to shikimate-pathway aromatic biosynthesis	4-Coumarate	11.5% (C mol/C mol) yield from adipic acid	Aromatic platform chemicals, flavor and fragrance precursors, polymer building blocks	8.8–9.8 M	Op de Hipt et al. (2025)
Aromatic / dicarboxylate node	Two-strain *Escherichia coli* consortium; *Rhodococcus* sp.; *Pseudomonas putida* KT2440	PBAT-derived terephthalic acid	Modular TPA catabolism; TPA transporter; strain 1 (TPA → PCA) and strain 2 (PCA → GA via mutant p-hydroxybenzoate hydroxylase)	Protocatechuate; gallic acid	Gallic acid at 92.5% molar yield	Antioxidants, antimicrobials, nutraceuticals, pharmaceutical intermediates, cosmetic preservatives, alkyl gallates, polymer and coating additives	PCA 14–15 M; GA 90–115 M	Hosaka et al. (2013)
Aromatic / dicarboxylate node	Engineered *Escherichia coli* (single-strain or two-strain)	TPA-derived protocatechuate/catechol	PCA decarboxylation module plus catechol hydroxylation module (PhKLMNOPQ)	Pyrogallol; *cis,cis*-muconic acid	n.r. (synthetic pathway demonstrated)	Polymer additives, antioxidants, adipic-acid precursor (via *cis,cis*-muconate), specialty aromatics	ccMA 120–150 M	Wang T. et al. (2018), Johnson et al. (2016) and Han et al. (2015)
Aromatic / dicarboxylate node	Engineered *Pseudomonas taiwanensis* (mixed-culture configuration)	PBAT/PBS-derived adipic acid + 1,4-butanediol	Tyrosine ammonia-lyase module; mixed-culture co-utilization of both monomers	4-Coumarate	~3 mM; 14.0% (C mol/C mol) combined yield (BDO 14.4%, AA 11.5%)	Aromatic building blocks, flavor and fragrance precursors, antioxidant precursors	8.8–9.8 M	Op de Hipt et al. (2025)
Consortium strategy	Enzymatic PET depolymerization coupled with engineered microbes	PET-derived monomers (TPA, EG) benchmark system	Two-stage enzyme-microbe architecture: depolymerization followed by engineered microbial conversion	Repolymerized PET / aromatic intermediates	Repolymerization demonstrated; depolymerization efficiency quantified	Benchmark proof-of-concept for integrating polymer breakdown with biosynthesis	35–40 B (PET)	Tournier et al. (2020)
Consortium strategy	Engineered *Escherichia coli*–*Pseudomonas putida* synthetic consortium	Mixed sugars; lignocellulosic hydrolysate	Division of labor, distributing substrate conversion and biosynthesis across specialized strains	Medium-chain-length PHA (mcl-PHA)	1.30 g L^−1^ (3% g/g) from mixed sugars; 1.02 g L^−1^ (2% g/g) from lignocellulosic hydrolysate	Flexible packaging, elastomeric and biomedical materials, polymer blends, surfactants	0.10–0.12 B	Qin et al. (2022)
Consortium strategy	Rationally designed engineered two-member consortia (synthetic ecology)	Mixed bioplastic hydrolysates	Reduced per-strain heterologous burden; redox compartmentalization; specialized detoxification; nutrient-partitioned population stability	Diverse (consortium-dependent)	Stable population ratios sustained over extended cultivation	Robust mixed-feedstock upcycling pipelines	n.r.	Ziesack et al. (2019) and Kerner et al. (2012)
Consortium strategy	Open / defined mixed microbial cultures	Heterogeneous biodegradable plastic and agro-industrial side streams	Non-aseptic operation; integration with anaerobic digestion and existing biorefinery/wastewater infrastructure	Volatile fatty acids; short-chain carboxylates	n.r. (industrially mature; lower product specificity)	Low-cost valorization of heterogeneous waste streams; volatile-fatty-acid platforms	n.r.	Hakalehto et al. (2025), Khedkar et al. (2025) and den Boer et al. (2016)

Systems are grouped by the central metabolic entry node at which plastic-derived monomers are funneled. Reported performance is given as stated in the cited sources; titers in g L^−1^, yields on the basis reported by the original authors. Host and organism names are italicized; n.r., not reported. Indicative market sizes refer to the global product class (USD; B, billion; M, million) and are not specific to plastic-derived material.

## 5 Techno-economic and life-cycle considerations

The metabolic strategies described above establish biochemical feasibility for biodegradable plastic upcycling. However, translation to circular implementation requires evaluation beyond pathway performance. TEA and LCA determine whether engineered depolymerization and bio-funneling can compete with conventional disposal, incineration, or fossil-derived production. In this context, TEA and LCA are not auxiliary analyses but system-level validation tools that determine whether circular bioconversion reduces environmental burden while remaining economically viable.

### 5.1 Life-cycle assessments

Life-cycle assessments of bioplastics primarily evaluate greenhouse gas emissions (GGE), global warming potential (GWP), eutrophication, acidification, and toxicity across production and end-of-life stages. Comparative LCAs consistently report that bio-based polymers such as PLA, PHA/PHB, and PBAT exhibit lower GWP and fossil carbon intensity than petroleum-derived plastics (Fonseca et al., 2023; Munagala et al., 2021; Anjana et al., 2023; Luo et al., 2024). However, reductions in climate metrics are frequently offset by higher impacts in freshwater and marine acidification, eutrophication, and human toxicity categories (Fonseca et al., 2023; Munagala et al., 2021; Anjana et al., 2023).

These trade-offs are largely attributable to upstream agricultural inputs and downstream processing stages. Feedstock cultivation often depends on fertilizer use and land transformation, while industrial side-stream feedstocks may require auxiliary chemicals to achieve competitive yields (Fonseca et al., 2023; Anjana et al., 2023). Substitution with more sustainable feedstocks such as sugarcane bagasse or sugar-beet pulp has been shown to reduce acidification and toxicity impacts, particularly when metabolic routes are redesigned to minimize auxiliary reagent requirements (Anjana et al., 2023; Ioannidou et al., 2022).

End-of-life scenarios further influence LCA outcomes. Although composting and incineration with energy recovery are often considered lower-impact options relative to landfilling, multiple studies indicate that recycling and upcycling pathways yield substantially lower environmental burdens across most impact categories (Hottle et al., 2017). However, few LCAs currently evaluate integrated bio-upcycling systems. Where assessed, environmental performance is constrained by low degradation rates, modest product yields, and significant energy and water inputs required to maintain bioreactor conditions (Yu et al., 2025; Anjana et al., 2023). In regions such as the United States, fossil-derived electricity and heat further limit net climate benefits.

Improvements in enzyme efficiency, metabolic flux distribution, toxin tolerance, and reduced feedback inhibition could substantially alter LCA outcomes by increasing carbon conversion efficiency and lowering residence time in bioprocessing systems. However, achieving near net-zero performance will also require parallel shifts in infrastructure and energy sourcing (Bishop et al., 2022). Thus, LCA data reinforces a key design principle emphasized throughout this review: biochemical feasibility alone is insufficient; process integration and system-level optimization ultimately determine sustainability.

### 5.2 Ecotoxicological comparison of degradation vs. upcycling intermediates

To provide a multidimensional toxicological comparison relevant to biodegradable plastic degradation and engineered upcycling systems, three acute hazard endpoints reported by the US Environmental Protection Agency (EPA) were evaluated independently: aquatic invertebrate toxicity (*Daphnia magna* 48 h EC_50_/LC_50_, mg/L), aquatic vertebrate toxicity (fish 96 h LC_50_, mg/L), and mammalian acute toxicity (rat oral LD_50_, mg/kg). This approach avoids inappropriate inter-species comparisons while allowing structured hazard contextualization. Across aquatic invertebrate, aquatic vertebrate, and mammalian acute toxicity endpoints, most biodegradable polyester monomers (lactate, succinate, adipate, and diols) fall within low-to-moderate acute toxicity ranges under standard test conditions (Table 4). In contrast, certain aromatic intermediates, such as catechol, exhibit comparatively lower LC_50_/EC_50_ values, indicating higher acute hazard potential if allowed to accumulate. Importantly, engineered bio-upcycling systems are designed to minimize intermediate buildup by tightly coupling depolymerization to downstream metabolic assimilation. Therefore, while intrinsic hazard profiles vary among compounds, environmental risk in circular bioprocesses is governed primarily by exposure control, metabolic flux management, and process containment rather than by inherent compound toxicity alone. This distinction reinforces the advantage of engineered upcycling over uncontrolled environmental degradation in reducing transient exposure to higher-hazard intermediates while enabling carbon valorization.

**Table 4:** Reported aquatic and mammalian toxicity of the principal monomers released during depolymerization of biodegradable plastics.

Monomer	Process context	Daphnia, 48 h EC₅₀ / LC₅₀ (mg L^−1^)	Fish, 96 h LC₅₀ (mg L^−1^)	Rat, oral LD₅₀ (mg kg^−1^)	Relative hazard
Lactic acid	PLA depolymerization monomer	130	>130	3,730	Low
Succinic acid	PBS monomer; central metabolic intermediate	>100	>100	2,260	Low
Adipic acid	PBAT and PBS diacid monomer	85.6	97	5,560	Low to moderate
1,4-Butanediol	PBS and PBAT diol monomer	881–2,650	>100	1,525	Low to moderate
Terephthalic acid	PBAT aromatic monomer	>982	>100	1,960	Low
Ethylene glycol	Polyester diol monomer	46,300	>10,000	4,700	Low

Values are representative acute-toxicity endpoints compiled from publicly available ecotoxicological and safety data reported by the US Environmental Protection Agency (EPA). EC₅₀, median effective concentration; LC₅₀, median lethal concentration; LD₅₀, median lethal dose. Higher values indicate lower toxicity. Values reported as “>” denote no effect observed up to the stated concentration. Endpoints are not directly comparable across species or exposure routes and are intended only to indicate relative hazard.

### 5.3 Techno-economic analysis

Techno-economic analyses of microbial conversion processes reveal a consistent pattern: systems that utilize purified plastic-derived monomers often underperform relative to processes operating on glucose-rich or biomass-derived media. Several reported studies lacked complete details on culture conditions or consistent unit reporting, limiting cross-comparison. Nevertheless, productivity rates in optimized laboratory systems approach those observed in industrial bioreactors when aeration and mixing are simulated at scale.

Processes converting lactate to pyruvate-derived chemicals or short-chain fatty acids (e.g., butyrate, propionate) remain comparatively underexplored. This gap may represent a strategic opportunity for engineered microbes to capture emerging markets for short-chain carboxylates, particularly if feedstock costs can be minimized by integrating with PLA hydrolysate streams.

A critical limitation identified across TEA studies is scale dependency. Many reported titers are achieved under laboratory conditions without energy integration or continuous processing. Industrial deployment will require demonstration under fed-batch or continuous bioreactor operation (100–100,000 L capacity), where volumetric productivity and downstream purification costs become dominant determinants of economic viability. It is vital to understand the scaling effects of fermentation, often encountered, to achieve a techno-economically feasible production yield, titer, and rate (Poddar and Scown, 2025).

**Figure 6:**
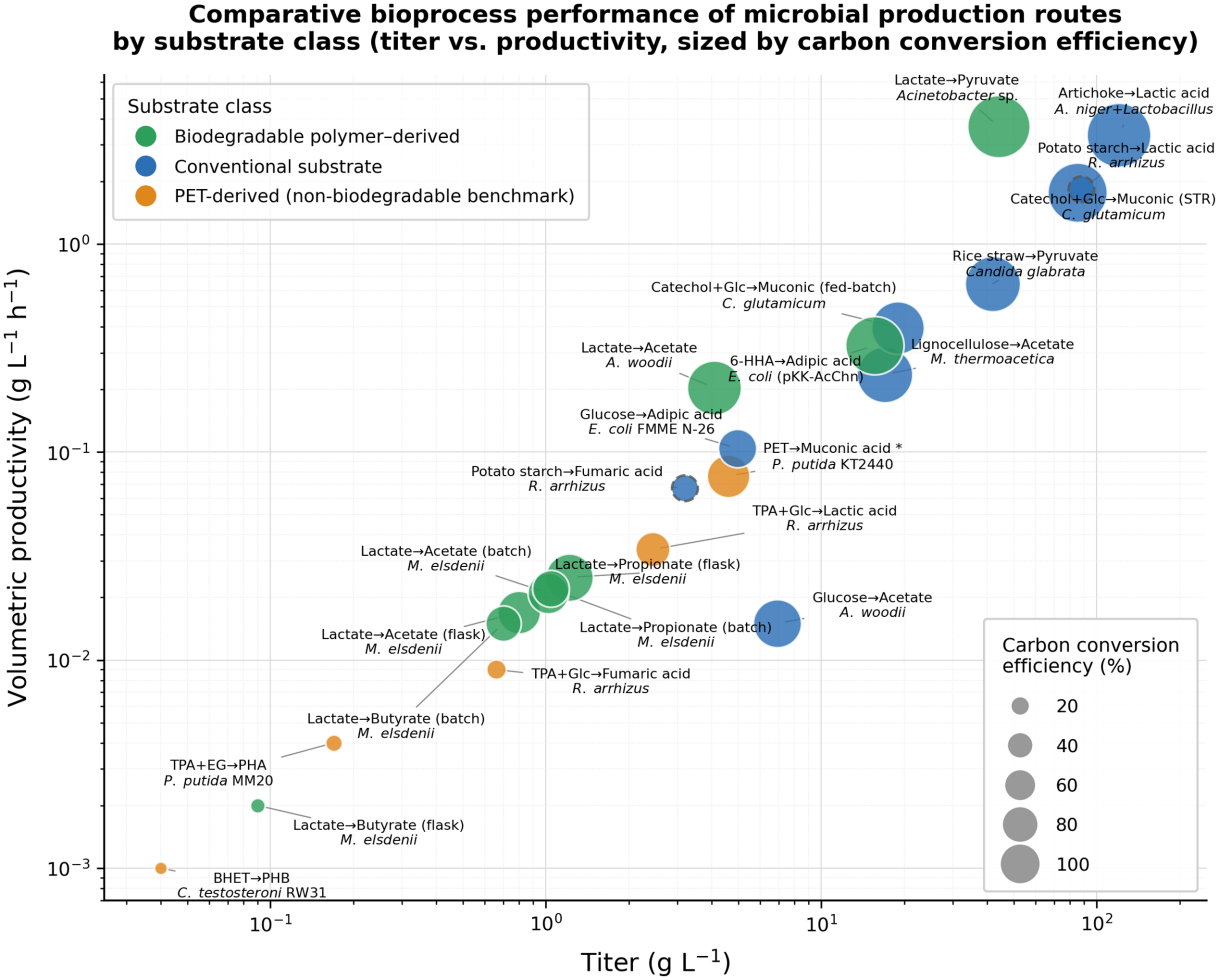
Comparative performance of microbial production routes from biodegradable polymer-derived intermediates, conventional substrates, and PET-derived benchmarks. Scatter plot of reported titers (g L^−1^), productivities (g L^−1^ h^−1^), and carbon efficiency/ recovery (%) for representative microbial production systems. Substrates were categorized as (i) biodegradable polymer–derived intermediates (green), (ii) conventional refined substrates (blue), and (iii) PET-derived intermediates used as non-biodegradable benchmarks (orange). Axes are shown on logarithmic scales to visualize performance differences across orders of magnitude. Specific data sources for each entry are provided in Table 5. Markers with a dashed edge denote entries for which carbon conversion efficiency was not reported and are shown at a fixed marker size.

To contextualize techno-economic feasibility, we compiled experimentally reported titers, productivities, and carbon conversion efficiencies across three substrate classes: biodegradable polymer-derived intermediates, conventional substrates, and PET-derived benchmarks in Table 5 and Figure 6. The resulting performance landscape reveals several systematic trends.

**Table 5:** Comparative carbon efficiency and bioprocess performance for the microbial conversion of biodegradable-plastic-derived, PET-derived, and conventional substrates.

Product	Substrate category	Substrate	Microbial strain	Cultivation mode	Carbon efficiency /recovery (%)	Titer (g L^−1^)	Productivity (g L^−1^ h^−1^)	References
Pyruvate	Biodegradable-polymer-derived	Lactic acid	*Acinetobacter* sp.	Flask	89.9	44.03	3.669	Ma et al. (2003)
Pyruvate	Conventional substrate	Rice straw	*Candida glabrata*	Flask	70	41.94	0.645	Pant and Pant (2025)
Acetate	Biodegradable-polymer-derived	Lactic acid	*Megasphaera elsdenii*	Flask	40.4	0.80	0.017	Prabhu et al. (2012)
Propionate	Biodegradable-polymer-derived	Lactic acid	*Megasphaera elsdenii*	Flask	50.6	1.22	0.025	Prabhu et al. (2012)
Butyrate	Biodegradable-polymer-derived	Lactic acid	*Megasphaera elsdenii*	Flask	2.1	0.09	0.002	Prabhu et al. (2012)
Acetate	Biodegradable-polymer-derived	Lactic acid	*Megasphaera elsdenii*	Batch	36.7	1.02	0.021	Prabhu et al. (2012)
Propionate	Biodegradable-polymer-derived	Lactic acid	*Megasphaera elsdenii*	Batch	29.3	1.04	0.022	Prabhu et al. (2012)
Butyrate	Biodegradable-polymer-derived	Lactic acid	*Megasphaera elsdenii*	Batch	27	0.70	0.015	Prabhu et al. (2012)
Acetate	Conventional substrate	Lignocellulosic sugars	*Moorella thermoacetica* ATCC 39073	Batch	71	17.0	0.236	Ehsanipour et al. (2016)
Acetate	Conventional substrate	Glucose	*Acetobacterium woodii*	Batch	52	6.92	0.015	Karekar et al. (2019)
Acetate	Biodegradable-polymer-derived	Lactic acid	*Acetobacterium woodii*	Batch	66.7	4.08	0.204	Schoelmerich et al. (2018)
cis,cis-Muconic acid	PET-derived benchmark	PET	*Pseudomonas putida* KT2440	Shake flask	40.4	4.6	0.0767	Liu et al. (2022)
cis,cis-Muconic acid	Conventional substrate	Catechol + glucose	*Corynebacterium glutamicum*	Fed-batch	63	19.0	0.396	Becker et al. (2018)
cis,cis-Muconic acid	Conventional substrate	Catechol + glucose	*Corynebacterium glutamicum*	Stirred-tank reactor	81	85.0	1.771	Becker et al. (2018)
Adipic acid	Biodegradable-polymer-derived	6-Hydroxyhexanoic acid	*Escherichia coli* (pKK-AcChn)	Fed-batch	78	15.6	0.325	Oh et al. (2024)
Adipic acid	Conventional substrate	Glucose	*Escherichia coli* FMME N-26	Bioreactor	32	4.97	0.104	Yuan et al. (2025)
Lactic acid	PET-derived benchmark	Terephthalic acid + glucose	*Rhizopus arrhizus*	Stirred-tank reactor	25	2.44	0.034	Cochereau et al. (2026)
Fumaric acid	PET-derived benchmark	Terephthalic acid + glucose	*Rhizopus arrhizus*	Stirred-tank reactor	6	0.66	0.009	Cochereau et al. (2026)
Lactic acid	Conventional substrate	Waste potato starch + rich medium	*Rhizopus arrhizus*	Stirred-tank reactor	n.r.	88.0	1.833	Zhang et al. (2009)
Fumaric acid	Conventional substrate	Waste potato starch + rich medium	*Rhizopus arrhizus*	Stirred-tank reactor	n.r.	3.2	0.067	Zhang et al. (2009)
Lactic acid	Conventional substrate	Jerusalem artichoke sugars	*Aspergillus niger* SL-09 + *Lactobacillus* sp. G-02	Fed-batch	94.5	120.5	3.35	Ge et al. (2009)
PHB	PET-derived benchmark	BHET	*Comamonas testosteroni* RW31	Shake flask	0.8	0.04	0.001	Molpeceres-García et al. (2025)
PHA	PET-derived benchmark	Terephthalic acid + ethylene glycol	*Pseudomonas putida* MM20 (pRK190)	Fed-batch	3.5	0.17	0.004	Manoli et al. (2024)

Substrates are grouped as biodegradable-polymer-derived, PET-derived benchmarks, or conventional substrates to allow direct comparison. Carbon efficiency/recovery is reported as stated in the cited sources; titer and volumetric productivity are given in g L^−1^ and g L^−1^ h^−1^, respectively. Host organism names are italicized; n.r., not reported. Values are not normalized between studies and reflect differing scales and cultivation modes.

First, biodegradable polymer-derived intermediates (e.g., lactic acid, 6-hydroxyhexanoic acid) cluster within productivity ranges comparable to conventional substrates, particularly at the pyruvate and acetyl-CoA nodes. For example, lactic acid-to-pyruvate conversion in *Acinetobacter* sp. achieved 44.03 g L^−1^ at 3.669 g L^−1^ h^−1^ with 89.9% carbon efficiency, closely approaching values obtained from rice straw-derived systems. Similarly, adipic acid production from 6-hydroxyhexanoic acid reached 15.6 g L^−1^ (0.325 g L^−1^ h^−1^, 78% carbon efficiency), exceeding glucose-based adipic acid production in both yield and productivity. These results indicate that once depolymerization has occurred, polymer-derived monomers can perform competitively at central metabolic nodes.

Second, PET-derived benchmarks generally occupy the lower-left quadrant of the titer-productivity space, reflecting lower carbon recovery and slower rates. For instance, PHB production from BHET and PHA production from TPA + EG exhibit both low titers and minimal productivity. This contrast reinforces the importance of upstream depolymerization chemistry and substrate assimilation efficiency in determining downstream process economics.

Third, conventional sugar-based systems still dominate the upper-right region of the performance map, particularly for lactic acid and *cis,cis*-muconic acid production under fed-batch or stirred tank conditions. However, these systems benefit from decades of strain and process optimization. When normalized by carbon conversion efficiency, several biodegradable-polymer-derived systems demonstrate competitive efficiency even at lower absolute titers, suggesting that metabolic routing, rather than substrate origin alone, governs carbon recovery (Table 5).

Importantly, the data also highlights node-specific constraints. At the pyruvate node, titers and productivities are generally high across substrate types, consistent with its central metabolic position. At the acetyl-CoA/β-oxidation node, performance varies depending on flux partitioning and redox balancing. At the aromatic node, yields remain comparatively lower, reflecting pathway length, regulatory complexity, and toxicity constraints. These node-dependent differences suggest that techno-economic optimization should prioritize improving flux density and tolerance at the aromatic interface rather than central metabolism.

Collectively, these comparative performance metrics suggest that metabolic feasibility has been largely demonstrated at the flask and pilot scales; the remaining techno-economic gap lies in process intensification, energy integration, and infrastructure compatibility rather than in fundamental biochemical limitations.

### 5.4 Economic barriers

Despite environmental advantages, biodegradable plastics remain cost-disadvantaged relative to petroleum-derived counterparts. Current PLA pricing (~US$2.19/kg) exceeds that of PET (~US$1.41/kg), PP (~US$0.96/kg), and BOPP (~US$1.41–2.09/kg). PHAs and PBAT exhibit even higher production costs (~US$4–6/kg and ~US$1.55/kg, respectively) (Luo et al., 2024).

These price differentials reflect feedstock variability, fermentation costs, purification requirements, and scale limitations. However, techno-economic risk assessments suggest that integration into existing biorefineries could substantially narrow this gap. For example, Monte Carlo simulation of PBS and PLA production from sugar-beet pulp indicated the potential to achieve parity with petroleum-based plastics under optimized conditions. Similar integration into sugarcane biorefineries for PBAT production demonstrated profitability even under 30% variation in total capital investment and operating costs (Ratshoshi et al., 2024).

Certain polymers, such as PCL, face structural pricing constraints due to medical-grade purity requirements (Yu et al., 2025; Kunamaneni, 2023). PHA production also encounters cost challenges associated with maintaining sterile conditions and preventing contamination during microbial cultivation. Thus, economic competitiveness will require both metabolic optimization and structural integration into existing industrial infrastructure.

### 5.5 Current technical bottlenecks and translational gaps

Although the preceding sections show that bio-upcycling of biodegradable plastics is biochemically feasible, translation to industrial practice is constrained by interlocking bottlenecks best assessed explicitly rather than implied. We summarize them here as open problems, since recognizing their current status is a prerequisite for prioritizing research effort.

Enzyme cost and reusability remain the dominant economic constraint. Depolymerase production is a major contributor to operating expenditure in enzymatic recycling, with reported enzyme prices spanning a wide range depending on expression strategy and production scale (Singh et al., 2021). High enzyme loadings are typically needed to overcome the crystallinity and morphological heterogeneity of post-consumer polyester, and reported catalytic efficiencies frequently obtained on amorphous or low-crystallinity model substrates substantially overstate performance on semi-crystalline, weathered material (Kaabel et al., 2021). Closing this gap requires not only more efficient and thermostable enzymes but realistic substrate benchmarking and effective enzyme immobilization or recycling.

Mixed waste-stream processing is a second barrier. Post-consumer biodegradable plastics arrive as heterogeneous mixtures of polymer types, additives, and contaminants, often co-mingled with conventional plastics. Selective depolymerization of chemically distinct ester linkages within such mixtures, for example, PLA/PBAT blends, is still immature, and feedstock variability undermines process standardization (Benninga et al., 2025; Xu et al., 2026).

Monomer purification imposes a third, often underappreciated, energy burden. Bio-recirculation to virgin-equivalent polymers requires polymerization-grade monomer purity, yet downstream recovery of organic and hydroxy acids from dilute fermentation broths remains energy-intensive at the moderate titers typical of plastic-derived feedstocks (Reifsteck et al., 2023).

Infrastructure compatibility and carbon efficiency complete the picture. Most current systems are validated only at flask or laboratory scale, placing the field at low-to-intermediate technology-readiness levels (Singh et al., 2021). Integration with existing waste infrastructure is constrained by the density overlap between several biodegradable polymers and conventional plastics, and by the fact that biodegradable plastics can contaminate conventional recycling streams. Comparing the two recycling logics directly, mechanical recycling is infrastructure-light and energy-efficient but progressively degrades polymer quality, whereas monomer-level bio-recirculation can regenerate virgin-equivalent material but carries higher enzyme, purification, and energy costs; the two are therefore complementary, with bio-recirculation best targeted at streams mechanical recycling cannot serve (Uekert et al., 2023). Taken together, these bottlenecks indicate that the remaining challenge is not biochemical feasibility but process robustness, energy integration, and system-level compatibility; the research priorities that follow are discussed in Section 6.7, Persistent Challenges and Research Priorities.

### 5.6 Marketability and integration into existing infrastructure

Given that complete displacement of petroleum plastics is unlikely, biodegradable polymers must leverage their unique advantages. PHAs, for example, comprise a diverse family of polymers with tunable mechanical and thermal properties. Continued exploration of novel synthesis pathways and strain engineering strategies may provide competitive differentiation in specialized markets. Their compatibility with food-contact applications and compostability position them favorably in packaging sectors aligned with national plastic pact commitments (Vidal et al., 2024).

Integration into existing infrastructure offers a practical pathway to economic viability. Biorefineries co-located with sugar mills have demonstrated feasibility for PBAT production via BDO intermediates, with sensitivity analyses supporting sustained profitability (Ratshoshi et al., 2024). Similar strategies have been proposed for PBS and PLA (Ratshoshi et al., 2021). Additionally, coupling PHA production with wastewater treatment has been modeled at 651 kg COD_PHA_·d^−1^ with production costs of EUR 0.11/kg COD_PHA_, while simultaneously reducing sludge handling burdens (Pozo-Morales et al., 2025). Although such systems increase energy demand and reduce biogas output, overall economic impact may remain favorable due to the biodegradability and downstream value of PHAs.

Beyond cost integration, biodegradable polymers carry application-specific advantages that conventional plastics cannot match, and that should be deliberately leveraged when defining circular-economy use cases. These include compostability for short-life food packaging and cutlery, and marine compatibility of selected PHA grades for fishing and aquaculture applications (Suzuki et al., 2021), biocompatibility for medical and surgical applications such as sutures, bone-fixation devices, and drug-delivery carriers (Koller, 2018; Yang et al., 2024), and time-controlled release behavior for agricultural mulch films and controlled-release fertilizers. Anchoring biodegradable polymers in these high-value, end-of-life-friendly niches rather than competing head-on with commodity polyethylene and polypropylene on price provides the economic gradient needed to sustain investment in upcycling infrastructure, while simultaneously reducing the volume of waste entering the upcycling stream.

End-of-life integration remains underdeveloped, in notable part due to the difficulty of sorting heterogeneous plastic waste. Gravity-based sorting for biodegradable plastics is commonly proposed but limited by density overlap (e.g., PCL and HDPE), leading to contamination risks (Taneepanichskul et al., 2022). Centrifugal, triboelectrostatic, and flotation sorting methods introduce additional chemical and energy demands; however, without clear economic incentives, infrastructure investment remains limited.

Upcycling into high-value chemicals provides one such incentive. For instance, *cis,cis*-muconic acid (projected market size US$38.7 million by 2031) has been produced from PET depolymerization (Globalinforesearch, 2025; Kolitha et al., 2023). Extending similar enzyme and metabolic engineering strategies to monomers derived from PLA, PBAT, PBS, and PHAs could substantially enhance value capture. Diversifying waste streams into specialty chemicals and advanced materials strengthens the economic logic of circular biorefineries.

Ultimately, the scalability of bio-upcycling will depend on coordinated advances in enzyme engineering, metabolic control, process intensification, infrastructure adaptation, and policy alignment. While environmental and biochemical feasibility are increasingly demonstrated, economic and systemic integration will determine whether circular bio-funneling transitions from proof-of-concept to industrial standard.

## 6 Future perspectives

The transition toward a circular bioeconomy for biodegradable plastics requires coordinated advances in enzymatic depolymerization, microbial bio-funneling, and integrated process design. While proof-of-concept studies demonstrate that PLA-, PHA-, PBAT-, and PBS-derived monomers can be biologically valorized, parallel progress in enzyme engineering and metabolic rewiring now suggests that structurally compatible synthetic polymers may also be routed through controlled depolymerization and microbial upgrading platforms. Translating these advances into scalable circular systems will require overcoming persistent technical, economic, and regulatory barriers across enzyme performance, strain robustness, process integration, and waste-stream heterogeneity.

### 6.1 Extending the circular bioeconomy framework to selected synthetic plastics

Beyond biodegradable polymers, engineered microbial systems are increasingly being applied to synthetic polyesters with hydrolysable ester linkages, enabling their integration into circular bioeconomy models. Rather than relying on uncontrolled environmental breakdown, polymers such as PET can be enzymatically depolymerized and fed into metabolic pathways for conversion into value-added products. For example, tandem enzymatic hydrolysis of PET followed by microbial assimilation has been demonstrated, where post-consumer PET is hydrolyzed to terephthalic acid and ethylene glycol and subsequently converted into polyhydroxyalkanoates (PHAs) or other bioproducts using engineered microbial chassis (Tiso et al., 2021). Engineered consortia have also been developed that divide labor between strains specialized in distinct monomer assimilation pathways, improving overall substrate assimilation and product formation from synthetic polymer hydrolysates (Bao et al., 2023). Recently, researchers demonstrated the conversion of PET to glycolic acid, which can be used in bio-based manufacturing (bioplastic) (Lin et al., 2026).

Integrating selected synthetic plastics into bio-upcycling pipelines expands feedstock flexibility and strengthens circularity by connecting traditionally linear waste streams to biologically driven valorization platforms. Advances in enzyme discovery and optimization for synthetic polyester depolymerization, together with synthetic biology and metabolic engineering to enhance monomer uptake and product synthesis, suggest that boundaries between biodegradable and compatible synthetic polymers may become defined more by engineered process design than by intrinsic chemical recalcitrance. This conceptual expansion highlights how engineered biological systems can function as programmable carbon routers in a unified circular materials economy, enabling both biodegradable and selected synthetic plastics to be transformed into renewable feedstocks for new polymers and high-value chemicals (Pardo et al., 2025).

### 6.2 Expanding microbial chassis diversity

There is a clear shift from exclusive reliance on model organisms toward microbial chassis better suited for biodegradable plastic depolymerization and monomer assimilation. Thermophilic microorganisms, including *Enterobacter* sp. and *Anoxybacillus rupiensis*, offer advantages for PLA and polyester hydrolysis at elevated temperatures, where increased polymer chain mobility enhances enzyme accessibility (Pham et al., 2024). Halophilic and marine-adapted systems may further expand deployment scenarios for PBS and PBAT residues in saline environments. Rather than broad pollutant degradation, future chassis development should prioritize metabolic compatibility with hydroxyacids, diacids, and diols derived from biodegradable polymers (Li et al., 2023). Microbial consortia represent a complementary strategy for biodegradable plastic upcycling by partitioning depolymerization and assimilation steps across specialized strains (Gregory et al., 2023; Zhang et al., 2023). Future work should move from empirical enrichment toward rationally designed consortia that couple polyester hydrolysis, hydroxy acid uptake, and product biosynthesis within controlled metabolic networks.

### 6.3 Integration of artificial intelligence and machine learning

Machine learning now plays a central role in accelerating the discovery of polyester-degrading enzymes relevant to PLA, PHA, PBAT, and PBS. Frameworks such as PED and PEZy-Miner integrate protein language models with substrate classification to identify candidate hydrolases with high predictive accuracy, reducing reliance on exhaustive screening (Jiang et al., 2023; Jiang et al., 2024). These tools are particularly valuable for identifying cutinases and esterases capable of targeting aliphatic-aromatic co-polyesters, where sequence-function relationships remain poorly defined. Computational protein redesign further enables stabilization and activity enhancement of polyester hydrolases under industrially relevant conditions. Although TurboPETase demonstrates the power of AI-guided engineering (Cui et al., 2024). Similar strategies can be extended to PLA and PBAT hydrolases, where improved thermostability, substrate promiscuity, and resistance to product inhibition remain critical gaps. Integrating deep learning with structure-guided mutagenesis may enable next-generation enzymes tailored specifically for biodegradable polymer recycling.

### 6.4 Digital twins and bioprocess optimization

Digital twin platforms integrating real-time sensor data with mechanistic and machine-learning models offer opportunities to optimize high-solids polyester hydrolysis and fermentation systems. For biodegradable plastics, such tools could dynamically adjust enzyme dosing, temperature, and feeding strategies in response to variable polymer crystallinity or monomer release rates (Alabi et al., 2025; Khan et al., 2024). In microbial bioreactors, predictive control could balance hydroxy acid uptake and downstream flux through pyruvate and acetyl-CoA nodes, improving stability under mixed-waste conditions.

### 6.5 Hybrid chemo-biological approaches

Hybrid chemo-biological systems may be particularly valuable for recalcitrant biodegradable co-polyesters such as PBAT and PBS. Mild catalytic pretreatment can reduce polymer molecular weight, increasing accessibility for microbial assimilation without compromising downstream bioconversion selectivity. Tandem chemo-microbial processes combining partial depolymerization with valorization have demonstrated efficient recovery of polyester-derived monomers and subsequent biological upgrading (Klauer et al., 2024; Li J. et al., 2024). For biodegradable plastics, such integration may minimize enzyme loadings and reduce total process cost while preserving carbon efficiency (Wang Y. et al., 2025b).

### 6.6 Biosafety, environmental impact, and risk–benefit analysis

Industrial deployment of engineered microbes for plastic upcycling raises legitimate environmental questions. Pulsed release of monomeric hydroxy acids (3-hydroxybutyrate, lactate, succinate) and aromatic intermediates (terephthalate, protocatechuate, catechol) from large-scale upcycling effluents could alter local microbial community composition through selective enrichment of acid-tolerant heterotrophs and PHA-degrading taxa, localized acidification affecting nitrification, and shifts in soil C:N stoichiometry where mulch-film hydrolysate is the dominant input (Boots et al., 2019). These ecological risks can be substantially mitigated by closed-loop recirculation of process water, neutralization polishing steps, and integration of upcycling streams into existing wastewater-treatment infrastructure rather than direct environmental discharge, ensuring that monomer release is contained within engineered process boundaries.

A second concern is horizontal gene transfer and persistence of engineered chassis in the environment. Although the metabolic genes used in upcycling are largely native to environmental bacteria and confer limited fitness advantage outside engineered conditions, transfer of selectable markers or unintended pathway combinations remains non-trivial. Validated containment options include synthetic auxotrophy on non-canonical amino acids (Mandell et al., 2015; Rovner et al., 2015), kill-switch circuits coupled to environmental cues (Chan et al., 2016; Rottinghaus et al., 2022), and physical containment via cell immobilization; field-relevant deployments should couple at least two orthogonal strategies (Lee et al., 2018). Occupational risks such as bioaerosols, hydrolase allergenicity, and chemobiological hazards fall within established BSL-1/2 industrial biosafety practice, but hybrid systems warrant integrated process hazard analyses.

These residual risks must be weighed against the relevant counterfactual, which is not “no plastic process” but the documented harms of the current linear plastic economy: microplastic accumulation in soils, freshwaters, and marine ecosystems; pollutants released by uncontrolled incineration; methane emissions from landfilled bioplastics; and emerging evidence of human dietary exposure to nano-plastics (Ziani et al., 2023). Set against these established harms, engineered upcycling offers reduced virgin-feedstock demand, controlled monomer recovery, and lower greenhouse-gas footprints at the cost of modest, reversible bioprocess risks. We therefore advocate a tiered deployment pathway, containing pilot operation, semi-contained industrial deployment within sealed biorefineries, and field-relevant release only after long-term Environmental Impact Assessment spanning at least one ecological cycle and including non-target organism testing, soil-microbiome metagenomics, and trophic-transfer studies, with this risk–benefit logic revisited iteratively as the technology matures.

### 6.7 Persistent challenges and research priorities

Despite advances in enzyme engineering and metabolic rewiring, several interdependent barriers continue to limit industrial implementation of biodegradable polyester upcycling. At the biological level, engineered strains must withstand chemical stress, osmotic stress, organic acid accumulation, fluctuating monomer release, and mixed hydrolysate compositions generated during depolymerization (Jiang and Bateer, 2025). Incomplete hydrolysis produces oligomers and transient intermediates that disrupt transport, redox balance, and membrane integrity, particularly at the aromatic node, where intermediate toxicity and regulatory bottlenecks constrain flux. Improving the chemical robustness of the host strain will require transporter optimization, dynamic pathway control, redox balancing, and adaptive evolution guided by multi-omics data. Indeed, the two-tier tolerance machinery, namely, detoxification and macromolecule protection, can be leveraged to design the strains (Jayakody et al., 2022; Jayakody and Jin, 2021; Jayakody et al., 2018).

Enzyme production remains a major cost contributor, accounting for ~20–30% of operating expenses in enzymatic recycling systems (Singh et al., 2021). High enzyme loading is often necessary to overcome polymer crystallinity and heterogeneous morphology. Industrial feasibility, therefore, depends on improving catalytic efficiency, thermostability, and enzyme recyclability, as well as on immobilization strategies or consolidated bioprocessing approaches that directly couple depolymerization to fermentation.

Process integration poses additional constraints. Efficient systems must prevent the accumulation of inhibitory oligomers while maintaining a stable pH and a sufficient supply of substrates. Separating hydrolysis and fermentation steps increases handling costs and carbon losses, whereas fully integrated systems introduce compatibility challenges between enzyme performance and microbial growth conditions.

At scale, physical and operational barriers become dominant. High-solids polyester slurries limit heat and mass transfer, increasing mixing energy demand and reducing effective enzyme-substrate contact (Rezaei et al., 2024). Downstream recovery of organic and hydroxy acids remains energy-intensive at moderate titers. Moreover, heterogeneous municipal waste streams introduce variability in polymer composition, additives, and contaminants, complicating feedstock standardization.

Reactor design offers concrete levers for addressing these stresses. High-cell-density fed-batch and continuous stirred-tank operation with dynamic dissolved-oxygen and pH control is standard for organic-acid and PHA fermentations and is directly transferable to hydroxy-acid-based upcycling streams (Koller, 2018). Bubble-column and airlift configurations further reduce shear and mixing-energy demand for high-solids polyester slurries (Bokelmann et al., 2025). Simultaneous saccharification and fermentation (SSF) and its membrane-coupled variant (SSFF) decouple optimal enzyme conditions from microbial growth, providing a direct template for consolidated polyester depolymerization-fermentation (Olofsson et al., 2008; Ishola et al., 2013).

Metabolic-node constraints identified earlier persist during scale-up. Pyruvate-derived systems are generally robust but sensitive to oxygen and redox balance. At the acetyl-CoA/β-oxidation node, competition with TCA entry and CoA availability limits secretion efficiency. At the aromatic node, pathway length, transcriptional regulation, and toxicity reduce carbon-conversion efficiency, thereby increasing substrate demand and purification burden.

Economic and infrastructure factors further influence feasibility. Many systems remain validated only at lab scale (flask); continuous operation, oxygen-transfer limitations, and long-term stability under mixed-substrate feeding remain under-characterized. Integration into existing waste management infrastructure requires compatibility with sorting, pretreatment, and regulatory frameworks governing waste-derived inputs.

**Figure 7:**
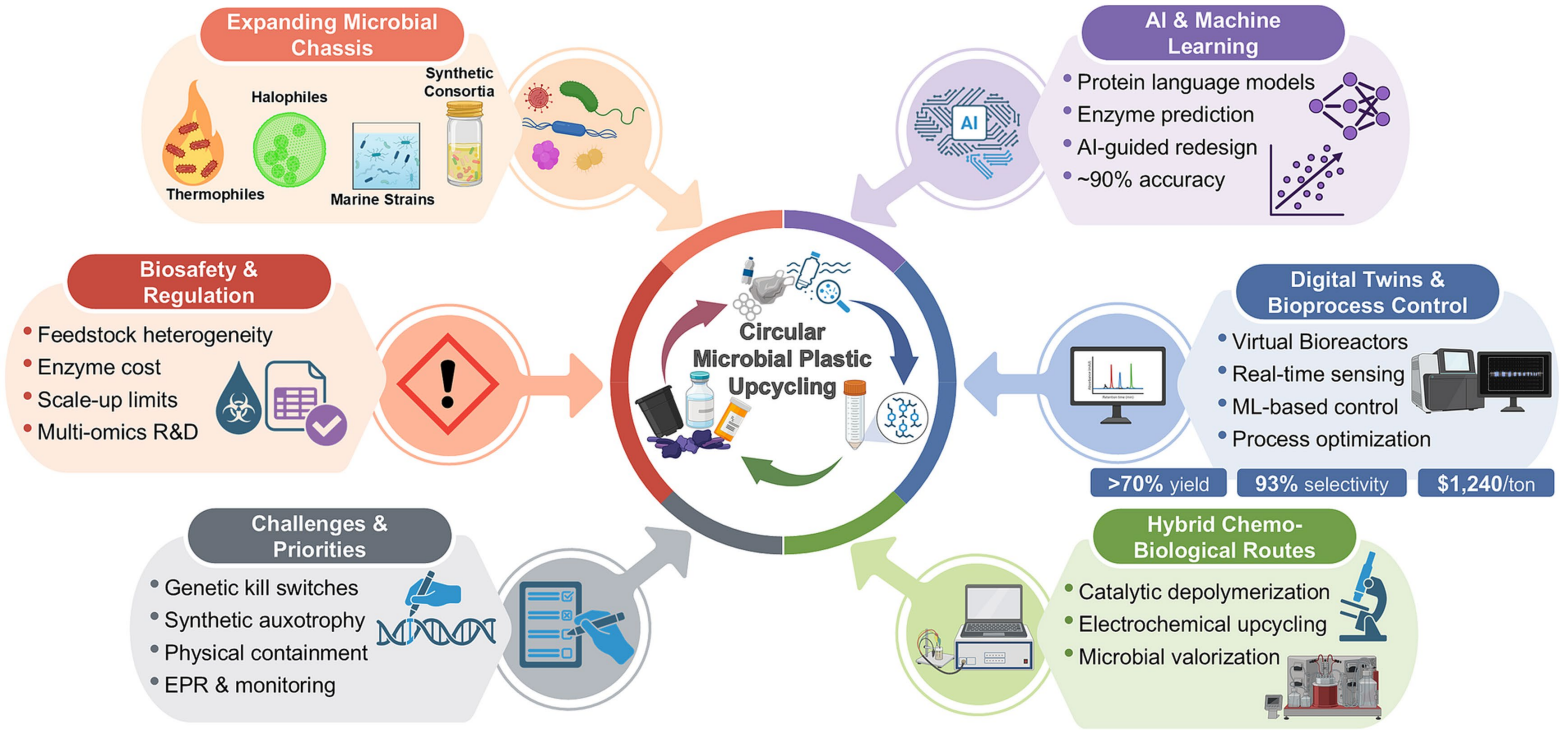
Overview of emerging strategies and future directions for microbial upcycling of biodegradable plastics. The figure illustrates key directions shaping future microbial plastic upcycling, including expansion of microbial chassis diversity, application of protein engineering and artificial intelligence to improve enzyme and pathway performance, use of digital twins for bioprocess monitoring and optimization, and integration of hybrid chemo-biological approaches for processing heterogeneous plastic waste streams. Practical considerations such as biosafety, regulation, scale-up constraints, and process economics are also highlighted, reflecting the challenges that must be addressed to enable closed-loop, circular valorization of biodegradable plastics.

Collectively, these challenges indicate that the remaining gap is not biochemical feasibility but process robustness and system integration. Priority areas include: (i) integrated depolymerization-fermentation platforms, (ii) tolerance engineering for mixed-monomer hydrolysates, (iii) process intensification and continuous operation, (iv) energy-efficient downstream recovery, and (v) pilot-scale validation under realistic waste variability (Dissanayake and Jayakody, 2021). Progress across these fronts will determine whether biodegradable polyester upcycling advances from laboratory demonstration to economically viable circular manufacturing. A consolidated overview of these emerging research directions and technological priorities for enabling circular microbial upcycling of biodegradable plastics is presented in Figure 7.

## 7 Conclusion

Biodegradable plastics were developed to mitigate the environmental persistence of conventional polymers, yet their real-world performance demonstrates that degradability alone does not ensure circularity. As outlined throughout this review, incomplete mineralization, infrastructure limitations, and uncontrolled environmental breakdown frequently result in carbon loss, microplastic accumulation, and secondary emissions. These limitations highlight a central premise of this work: true sustainability requires moving beyond passive biodegradation toward controlled, engineered carbon recovery.

Here, we presented advances in enzyme discovery, protein engineering, and metabolic pathway rewiring into a unified bio-funneling framework organized around three major metabolic nodes: pyruvate, acetyl-CoA/β-oxidation, and aromatic/dicarboxylate entry points. This node-based perspective reveals that biodegradable plastic-derived monomers are not terminal waste products but programmable carbon intermediates. Through targeted flux redirection, redox balancing, and dynamic regulation, engineered microbial systems can convert lactate, hydroxyalkanoates, succinate, adipate, butanediol, and terephthalate into platform chemicals, specialty monomers, fuels, and next-generation biopolymers. Importantly, many of these engineering principles extend beyond biodegradable plastics to selected synthetic polymers, enabling integration of heterogeneous waste streams into circular biomanufacturing platforms.

Enzyme engineering has emerged as a critical enabling technology, improving catalytic efficiency, thermostability, substrate scope, and tolerance to realistic waste conditions. Concurrently, advances in strain engineering, adaptive laboratory evolution, synthetic consortia design, and cell-free systems have expanded the range of assimilable monomers and attainable product spectra. Together, these innovations reposition plastic depolymerases from environmental cleanup tools to industrial biocatalysts embedded within regenerative value chains.

However, technological feasibility alone does not guarantee implementation. Techno-economic and life-cycle analyses indicate that enzyme cost, process integration, energy demand, substrate heterogeneity, and downstream separation remain key determinants of scalability. Moreover, industrial deployment requires robust strains capable of tolerating mixed monomer streams, osmotic stress, acid accumulation, and fluctuating feedstock composition. Addressing these barriers demands coordinated advances in high-throughput enzyme optimization, systems-level flux modeling, pilot-scale validation, and infrastructure alignment. Importantly, evaluation frameworks must consider not only greenhouse gas reduction but also toxicity mitigation, resource efficiency, and compatibility with existing waste management systems.

Collectively, the critical evaluation of green technology presented in this review supports a paradigm shift: biodegradable plastics should not be viewed merely as materials designed to disappear, but as renewable carbon reservoirs designed to circulate. By integrating enzymatic depolymerization, metabolic bio-funneling, and engineered valorization pathways, bio-upcycling platforms offer a route to actively close the loop on plastic carbon. The convergence of synthetic biology, computational protein design, systems metabolic engineering, and process integration now positions engineered microorganisms as central actors in the transition from a linear plastics economy to a circular bioeconomy. Ultimately, the future of sustainable plastics will depend not on how efficiently materials degrade in uncontrolled environments, but on how precisely their carbon can be captured, redirected, and regenerated. Engineered microbial systems provide the conceptual and technological foundation to achieve this green transformation. The central message of this review is that biodegradability and circularity are not the same goal; engineered microbial upcycling is the bridge that converts degradable material into a genuinely circular one.
